# Insights to improve the activity of glycosyl phosphorylases from Ruminococcus *albus* 8 with cello-oligosaccharides

**DOI:** 10.3389/fchem.2023.1176537

**Published:** 2023-04-07

**Authors:** Alem Storani, Sergio A. Guerrero, Alberto A. Iglesias

**Affiliations:** Laboratorio de Enzimología Molecular, Instituto de Agrobiotecnología Del Litoral (CONICET—UNL), Facultad de Bioquímica y Ciencias Biológicas, Universidad Nacional Del Litoral, Santa Fe, Argentina

**Keywords:** cellobiose phosphorylase, cellodextrin phosphorylase, cello-oligosaccharides, CBM, enzymatic catalysis

## Abstract

The phosphorolysis of cello-oligosaccharides is a critical process played in the rumen by *Ruminococcus albus* to degrade cellulose. Cellodextrins, made up of a few glucosyl units, have gained lots of interest by their potential applications. Here, we characterized a cellobiose phosphorylase (*Ral*CBP) and a cellodextrin phosphorylase (*Ral*CDP) from *R. albus* 8. This latter was further analyzed in detail by constructing a truncated mutant (*Ral*∆N63CDP) lacking the N-terminal domain and a chimeric protein by fusing a CBM (*Ral*CDP-CBM37). *Ral*CBP showed a typical behavior with high activity on cellobiose. Instead, *Ral*CDP extended its activity to longer soluble or insoluble cello-oligosaccharides. The catalytic efficiency of *Ral*CDP was higher with cellotetraose and cellopentaose as substrates for both reaction directions. Concerning properties of *Ral*∆N63CDP, results support roles for the N-terminal domain in the conformation of the homo-dimer and conferring the enzyme the capacity to catalyze the phosphorolytic reaction. This mutant exhibited reduced affinity toward phosphate and increased to glucose-1-phosphate. Further, the CBM37 module showed functionality when fused to *Ral*CDP, as *Ral*CDP-CBM37 exhibited an enhanced ability to use insoluble cellulosic substrates. Data obtained from this enzyme’s binding parameters to cellulosic polysaccharides agree with the kinetic results. Besides, studies of synthesis and phosphorolysis of cello-saccharides at long-time reactions served to identify the utility of these enzymes. While *Ral*CDP produces a mixture of cello-oligosaccharides (from cellotriose to longer oligosaccharides), the impaired phosphorolytic activity makes *Ral*∆N63CDP lead mainly toward the synthesis of cellotetraose. On the other hand, *Ral*CDP-CBM37 remarks on the utility of obtaining glucose-1-phosphate from cellulosic compounds.

## 1 Introduction

Cellulose, the quantitatively highest biopolymer on Earth, plays a critical role as a structural polymer, source of sugars and material for industrial applications (Moon et al., 2011; Huber et al., 2012; Nakajima M et al., 2017). Cellulose is a linear polymer of a hundred to a thousand glucosyl units linked through β-(1 → 4)-glycosidic bonds. Cellulose oligomers, or cellodextrins, made up of a few glucosyl units, have gained some interest because of their properties (Kluge et al., 2019; Ávila et al., 2021). These materials have potential applications for non-digestible dietary fiber products (Adharis et al., 2018), novel bio-based surfactants (Billès et al., 2016), hybrid nanomaterials (Enomoto-Rogers et al., 2010; 2011), and scaffold candidates for tissue engineering (Wang et al., 2017). Cellodextrins are interesting feed ingredients in livestock animals, showing potential prebiotic and health-promoting properties (Sybesma et al., 2015).

Cello-oligosaccharides have been produced from the degradation of natural cellulose or synthetic pathways via chemical or enzymatic reactions (Billès et al., 2017; Kluge et al., 2019). Soluble cellodextrins were proven to be beneficial dietary fibers and prebiotics that stimulate the growth of many healthy human gut bacteria more efficiently than inulin, trans-galacto-oligosaccharides, and cellobiose alone (Zhong et al., 2020). One way to generate cellodextrins is through chemical (e.g., acid-catalyzed cellulose degradation) or enzymatic hydrolysis of cellulose. However, there are important concerns about chemical degradation because of the generation of oligosaccharides and unwanted by-products. The enzymatic hydrolysis, meanwhile, requires highly pure enzymes (e.g., cellulases, β-glucosidases) and gives a mixture of cellodextrins (Zhong et al., 2019). Furthermore, the isolation of the cellodextrins from hydrolysis mixtures needs significant efforts in downstream processing (Zhang and Lynd, 2003). An alternative approach is to produce cellodextrins through bottom-up synthesis to overcome cellulose degradation issues. Since chemical synthesis involves multistep procedures, difficulties appear in controlling glycosidic bond formation’s regioselectivity and stereospecificity during polymerization and obtaining high molecular weight polysaccharides (Xiao and Grinstaff, 2017). Enzymatic routes constitute convenient tools for overcoming such critical troubles (Billès et al., 2017). *In vitro* enzymatic synthesis of cello-oligosaccharides provides some advantages compared to the previous methods. For example, well-controlled structures of products are obtained in a one-step polymerization owing to the high regio-, *enantio*-, chemo-, and stereo-selectivity of the enzymes. In addition, the production of controlled product composition eliminates the need for posterior purification steps (Zhong et al., 2020). Moreover, enzymes are non-toxic compounds that catalyze the reaction under ecologic environments, and they are isolated from sustainable resources or produced as recombinant proteins (Shoda et al., 2016; Fodor et al., 2017).

*Ruminococcus albus* 8 is a critical ruminal bacterium involved in the digestion of dietary cellulose. This microorganism degrades cello-oligosaccharides mainly through phosphorolysis catalyzed by cellobiose phosphorylase (*Ral*CBP; EC 2.4.1.2) and cellodextrin phosphorylase (*Ral*CDP; EC 2.4.1.49) rather than hydrolysis catalyzed by β-glucosidase (Hamura et al., 2012; Sawano et al., 2013). Glycoside phosphorylases belong to the glycoside hydrolase family 94 (GH94) and play an important role in the metabolism of long-chain cellodextrins (Ubiparip et al., 2021). They catalyze a glycoside’s reversible phosphorolysis to produce the corresponding sugar 1-phosphate (Eq. 1), enabling efficient synthesis of a diversity of oligosaccharides with sugar 1-phosphate as the donor and a suitable carbohydrate acceptor in the synthetic reaction with strict regioselectivity (Luley-Goedl and Nidetzky, 2010; Nakai et al., 2010; O’Neill and Field, 2015; Puchart, 2015; Zhong et al., 2019; Gabrielli et al., 2021).

$$Cello-{oligosacharide}_{\left(n\right)}+Pi\;↔Cello-{oligosacharide}_{\left(n-1\right)}+Glc-1P$$ (1)

Cellodextrin phosphorylases have been extensively used in the enzymatic synthesis of polysaccharides (Nakai et al., 2013; O’Neill and Field, 2015; Zhong et al., 2019; Zhong and Nidetzky, 2020; Nidetzky and Zhong, 2021). They have the broadest acceptor and donor substrate specificity of all GH94 enzymes, making them good tools for suitable biocatalytic production of diverse carbohydrates and related molecules. Still, the average degree of polymerization (DP) of cellodextrin produced by CDP ranged from 8 to 10 glucose units, suggesting that these enzymes cannot elongate high DP cellodextrin chains (Ubiparip et al., 2021). This lack of capacity may result from the decrease in cellodextrin solubility as their DP increases, leading to reduced access of the soluble enzyme to the longer substrates. To overcome this issue, enzymes from different GH families, usually contain a catalytic module and one or more non-catalytic carbohydrate-binding modules (CBMs) (Boraston et al., 2004). CBMs can enrich enzymes on the surface of solid substrates through affinity adsorption to enhance their activity (Shoseyov et al., 2006). Remarkably, in addition to the catalytic domain, most glycosyl hydrolases from *R. albus* display different motifs involved in substrate binding and/or cell adhesion (Christopherson et al., 2014; Storani et al., 2020; Yeoman et al., 2021). Among them, CBM from family 37 (CBM37), exclusive from this bacterium, exhibits an affinity to broad substrates like cellulose, xylans, and other oligosaccharides (Ezer et al., 2008). Unlike many glycoside hydrolases, no report deals with glycoside phosphorylase containing a CBM. From the crystal structure of *Clostridium thermocellum* CDP, it was identified that this protein had a unique N-terminal extension involved in homodimer assembly and contributed to the substrate binding site conformation (O’Neill et al., 2017). We identified that multiple GH94 enzymes have a very conserved N-terminal region that could be involved in homodimer assembly and/or binding site conformation.

The present work describes the structural and functional characterization of two members of the family GH94 (CDP and CBP) from *R. albus* 8. Here, we analyzed the putative function of the first 63 N-terminal amino acids conserved in *Ral*CDP as a putative CBM. On the other hand, we hypothesized that adding a CBM to a cellodextrin phosphorylase catalytic domain could improve enzyme access to insoluble substrates, thus enhancing its activity. To achieve this goal, we explored how the fusion of a CBM37_2 from *R. albus* 8 Cel5G affects its activity on insoluble substrates.

## 2 Materials and methods

### 2.1 Bacteria, plasmids, primers, and reagents

*Escherichia coli* Top 10 F′ and *E. coli* BL21 (DE3) (Invitrogen) served as hosts for cloning purposes and expression of the genes cloned into pRSFDuet and pETDuet vectors (Novagen). DNA manipulations, *E. coli* cultures, and transformations were performed according to standard protocols (Sambrook and Russell, 2001). Supplementary Table S1 details all the primers (obtained from GenBiotech) utilized in this work. Carboxymethyl cellulose (CMC), glucose-1-phosphate (Glc-1P), cellobiose (C2) and glucose (Glc) were from Sigma-Aldrich. Cellotriose (C3), cellotetraose (C4) and cellopentaose (C5) were from CarboSynth. All other reagents were of the highest purity available. Phosphoric acid swollen cellulose (PASC) was prepared from Cellulose Powder (ICN) after water slurring, cellulose dissolution in ortho-phosphoric acid, and regeneration in water (Percival Zhang et al., 2006). Insoluble PASC was prepared in concentrated phosphoric acid with partial hydrolysis in an ice bath for 1 h (Zhang and Lynd, 2004).

### 2.2 Construction of CDP variants

The DNA sequences coding for *Ral*CBP (GenBank accession number: OP866768) and *Ral*CDP (GenBank accession number: OP866769), as reported in the elucidated genome assembly from *R. albus* 8 (GCA_000178155.2), were codon-optimized for expression in *E. coli* based on RefSeq: WP_002848762.1 and RefSeq: WP_002848532.1, respectively. Sequences were *de novo* synthesized by Bio Basic (Canada) and provided in pUC57/*ralcdp* and pUC57/*ralcbp*, respectively. The synthetic genes were cloned into the pETDuet vector (Novagen) between the SacI and SalI sites to produce the recombinant proteins fused to a His_6_-tag at the N-terminus.

For CBM37 fusion, the DNA sequence coding for CBM37 from *R. albus* 8 Cel5G was amplified using primers FowCBM37 and RevCBM37, respectively (Supplementary Table S1), containing *Bam*HI and *Not*I restriction sites for cloning the PCR fragment into a designed cassette. This cassette allows cloning CBM37 to either N- or C- terminal of the previously cloned gene fused by a glycine linker (Gly X9) into pETDuet or pRSFDuet expression vectors between *Sac*I and *Sal*I restriction sites. To construct Ral∆N63CDP, the DNA region (first 189 nucleotides) coding for the putative CBM was removed by PCR from pUC57/*ralcdp*. Primers FowGHCDP and RevGHCDP (Supplementary Table S1), respectively containing *Sac*I and *Sal*I restriction sites, were used for cloning the PCR fragment into the pRSFDuet expression vector.

### 2.3 Protein expression and purification

The constructs [pETDuet/RalCBP] [pETDuet/RalCDP] [pRSFDuet/Ral∆N63CDP] and [pETDuet/RalCDP-CBM37] were used to transform *E. coli* BL21 (DE3) cells (Invitrogen). To produce the recombinant proteins, we inoculated 1 L of LB medium (supplemented with 50 μg mL^−1^ kanamycin or ampicillin) with a 1/100 dilution of an overnight culture. Cells were grown at 37°C and 180 rpm in an orbital shaker until OD_600nm_ was ∼0.6. Gene expression was induced with the addition of 0.2 mM isopropyl-β-D-1-thiogalactopyranoside and further growing at 22°C overnight. The cells were harvested by centrifugation at 5,000 ×g at room temperature for 15 min and kept at −20°C until use.

The cell pellet was suspended in 25 mL of Buffer A [25 mM Tris-HCl pH 8.0, 300 mM NaCl, 5% (v/v) glycerol, 10 mM imidazole], and disrupted by sonication. The resulting suspension was centrifuged twice at 20,000 ×g at 4°C for 10 min. The supernatant was loaded onto a 1 mL HisTrap column connected to an ÄKTA Explorer 100 purification system (GE Healthcare), previously equilibrated with Buffer A. After washing the column with 10 mL of Buffer A, the recombinant protein was eluted with a linear gradient of imidazole (10–300 mM, 50 mL). The fractions containing the enzymes of interest were collected and concentrated with Amicon™ Ultra Centrifugal Filter (Merk). The enzyme pool was loaded onto a HiLoad 16/600 Superdex 200 column (GE Healthcare), previously equilibrated with Buffer G [50 mM HEPES-NaOH pH 8.0, 100 mM NaCl, 0.1 mM EDTA]. The pool of the active fractions was concentrated, and protein preparations were supplemented with 5% (v/v) glycerol and stored at −80°C until use. Under these conditions, the purified enzymes remained stable and active for at least 6 months.

### 2.4 Native molecular mass determination

The native molecular mass of the purified recombinant proteins was determined by using a Superdex 200 10/300 column (GE Healthcare), equilibrated with Buffer G. A calibration curve was constructed by plotting the Kav values *versus* log10 of the molecular mass of standard proteins (ribonuclease, 13.7 kDa; carbonic anhydrase, 29 kDa; ovalbumin, 43 kDa; conalbumin, 75 kDa; aldolase, 158 kDa; ferritin, 440 kDa; and thyroglobulin, 669 kDa). The Kav was calculated as (V_e_-V_0_)/(V_t_-V_0_), where V_e_ is the elution volume of the protein, V_0_ is the elution volume of Dextran Blue, and V_t_is the total volume of the column.

### 2.5 Enzyme activity assay and determination of kinetic constants

Cellobiose and cellodextrin phosphorylase activities were determined for phosphorolysis and synthetic directions. The reaction of cello-oligosaccharides synthesis was measured following the inorganic phosphate (Pi) released from the glucosyl donor using a highly sensitive colorimetric method (Fusari et al., 2006). The standard reaction media contained 100 mM sodium acetate buffer pH 6.0, 2 mM Glc-1P, 20 mM of different glucosyl acceptors (glucose, cellobiose, cellotriose, cellotetraose or cellopentaose), and a proper dilution of the enzyme in a final volume of 50 μL. Reactions were incubated for 10 min at 45°C and terminated with the addition of the Malachite Green reagent. The complex formed with Pi was measured at 630 nm in a Multiskan GO microplate reader (Thermo Scientific). To determine phosphorolytic activity, the reaction mixture (50 μL) contained 50 mM sodium acetate buffer (pH 6.0), 20 mM cellobiose, 50 mM Pi (potassium salt), and an appropriate amount of enzyme. After 10 min incubation at 45°C, the reaction was stopped by heating at 100°C for 10 min. The glucose oxidase-peroxidase method measured D-glucose liberated from cellobiose (Ambade et al., 1998).

When an oligosaccharide (*n* > 2) was used as a substrate, arsenolysis was measured instead of phosphorolysis by incubating the substrate (20 mM or 10 mg/mL) in a reaction mixture of 50 μL containing 50 mM sodium acetate buffer (pH 6.0), 50 mM arsenate (sodium salt) and a proper enzyme dilution for 10 min at 45°C (Eis and Nidetzky, 1999). D-glucose released from arsenolysis (since glucose 1-arsenate is unstable) was determined by the glucose oxidase-peroxidase method (Ambade et al., 1998). Comparative determination between phosphorolysis and arsenolysis reactions was performed with cellobiose as substrate (Supplementary Figure S1).

In all the assays, the substrate consumption was maintained below 10% as a control to guarantee the proper determination of initial velocity (*v*_o_). One unit (U) of enzyme activity equals 1 μmol of glucose/Pi released per minute under the respective assay conditions specified above.

Kinetic assays were performed using specified concentrations and conditions for all reaction mixture components. Saturation curves were carried out by assaying the respective enzyme activity at the saturating level of a fixed substrate and different concentrations of the variable substrate. The experimental data were plotted as enzyme activity (U.mg^-1^) *versus* substrate concentration (mM or mg/mL), and kinetic constants were determined by fitting the data to the Michaelis-Menten equation (Eq. 2). *k*_cat_ was calculated with the enzyme concentration ([E]) from *V*_max_ as *k*_cat_ = *V*_max_/[E]. All kinetic constants are the mean of at least three data sets, reproducible within ±10%.

$$v_{o=\frac{V_{\mathrm{Max}}\left[S\right]}{K_{M}+\left[S\right]}}$$ (2)

### 2.6 Carbohydrate binding assays

Enzymes’ binding capacity to cellulosic polysaccharides was determined as follows. The enzyme (10 µM) was mixed with 5 mg PASC in 0.25 mL of 50 mM sodium acetate buffer (pH 6.0) and incubated at 25°C for 1 h with stirring (1,500 x*g*). Stability control was performed by setting the protein in the same conditions without the polysaccharide. After centrifugation at 5,000 x*g* for 5 min to precipitate the polysaccharide, the supernatant was separated, and the unbound enzyme was quantified by the Bradford procedure (Bradford, 1976). The insoluble polysaccharide was washed thrice with sodium acetate buffer; then bound protein was eluted with 0.25 mL sodium dodecyl sulfate (SDS) 10% (w/v) at 90°C for 10 min and analyzed by SDS–polyacrylamide gel electrophoresis (SDS-PAGE).

Assays quantifying the capacity of enzyme binding to saccharides were carried out with 0.3–10 µM protein with 5 mg of polysaccharide in 0.25 mL of 50 mM sodium acetate buffer (pH 6.0). The maximum adsorption capacity (*A*_max_) and dissociation constant (*K*_d_) were determined by regressing binding isotherm data to a modified Langmuir-type binding model as described previously (Hong et al., 2007).

### 2.7 Polysaccharides synthesis and phosphorolysis

The analysis of cello-oligosaccharides synthesized by carrying out reactions (triplicated) at 45°C under agitation at a rate of 800 rpm in a Thermo Mixer HCM100-Pro (DLab Scientific). The reaction mixture contained 10 mM cellobiose, 100 nM purified enzyme in sodium acetate buffer (50 mM, pH 6.0) and different Glc-1P concentrations (0, 10, 30 and 50 mM) in a total volume of 0.05 mL. The reactions were stopped at different times by incubating the mixture at 100°C for 10 min. The samples were centrifuged at 20,000 xg, and the supernatant was separated from the pellet. The supernatant was analyzed for soluble cellodextrins using thin-layer chromatography (TLC) with a mobile phase of ethyl acetate: acetic acid: water (4:3:3, by volume). Staining was performed with orcinol 0.25% reagent in ethanol: sulfuric acid solution (95:5, v/v) at 95°C.

The soluble oligosaccharides were additionally analyzed by high-performance liquid chromatography (HPLC) on a Shimadzu LC-2050 system (Merck, Darmstadt, Germany) using a Luna Omega 3 μm NH2 column (100 Å, 150 × 4.6 mm; Phenomenex, Aschaffenburg, Germany) operated at 30°C. Acetonitrile: water (65: 35, by volume) was used as eluent at a flow rate of 1 mL/min. Refractive index detection was used to quantify cello-oligosaccharides and cellobiose. Calibration was performed with cellobiose, cellotriose, cellotetraose, and cellopentaose as standards.

The capacity for deconstructing high DP cello-oligosaccharides of the enzyme was measured. PASC arsenolysis was performed in a reaction mixture of 50 μL containing 1 mg/mL PASC, 50 mM sodium acetate buffer pH 5, 100 mM sodium arsenate, and 5 nM enzyme. The glucose oxidase-peroxidase method (Ambade et al., 1998) determined D-glucose released from arsenolysis. PASC phosphorolysis was performed in the same conditions (a reaction mixture of 50 μL containing 1 mg/mL PASC, 50 mM sodium acetate buffer pH 5, 50 mM Pi, and 5 nM enzyme), but Glc-1P, released from the reaction, was determined enzymatically using a continuous coupled enzyme assay (Eis and Nidetzky, 1999).

### 2.8 Other assays

Protein concentration was determined following the procedure described by Bradford (Bradford, 1976), using BSA as the standard. Polyacrylamide gel electrophoresis was carried out under denaturing conditions (SDS-PAGE) to determine the purity of the enzymes (Laemmli, 1970). The average DP of PASC and CMC was determined by exhaustive hydrolysis by phenol-sulfuric method and determining reducing ends by Somogyi-Nelson (Zhang and Lynd, 2005).

## 3 Results

### 3.1 Sequence alignment and structure analysis

Cellobiose and cellodextrin phosphorylases belong to family GH94 together with other glycosyl phosphorylases like laminaribiose phosphorylases (LBPs), chitobiose phosphorylases (ChBPs), and cellobionic acid phosphorylases (CBAPs). They are commonly homodimers, and each subunit is composed of three distinct domains: an N-terminal β-sandwich domain that forms most of the dimer interface, a helical linker, and an (α/α)_6_-barrel catalytic domain (Hidaka et al., 2004, 2006; Bianchetti et al., 2011; Nakajima H et al., 2017; Kuhaudomlarp et al., 2019). Interestingly, the length and conformation of catalytic, adjacent, and opposite loops were identified as the determining features that restrict the size of the acceptor binding site differentiating between CBPs, CDPs, and ChBPs (Hidaka et al., 2004; 2006; De Groeve et al., 2010; Bianchetti et al., 2011; O’Neill et al., 2017; Gabrielli et al., 2021).

**FIGURE 1:**
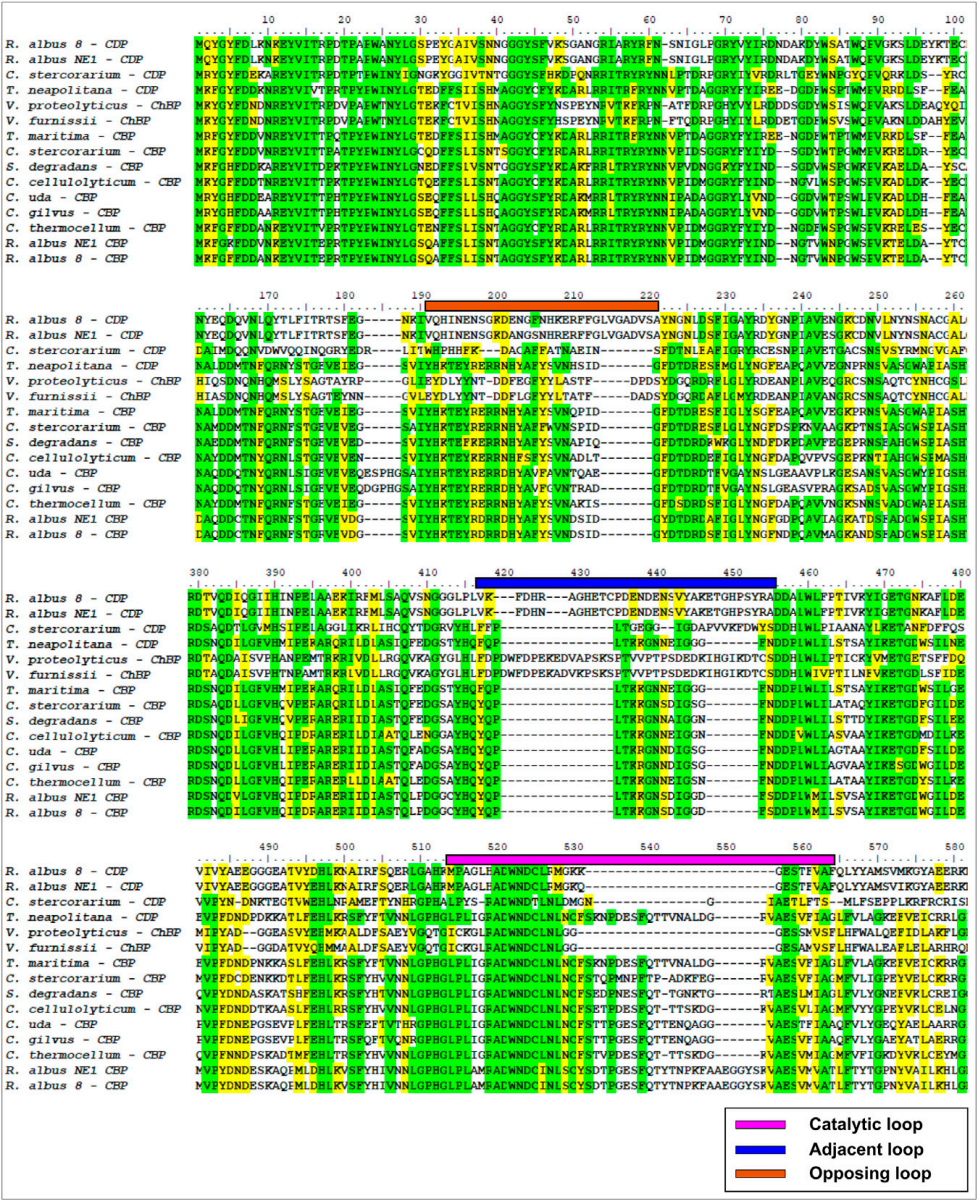
Multiple alignment of partial sequences from GH94 family enzymes. Color lines over the sequences delimit opposite (orange), adjacent (blue), and catalytic (magenta) loops.

We recognized two putative glycosyl phosphorylases from the GH94 family in the proteome of *R. albus* 8 project ID UP000004259, identified with entry codes E9SB92 and E9SAK5. Protein sequence E9SB92 is entirely identical to CBP from *R. albus* NE1 (Hamura et al., 2012). It presents high identity (67% and 56%, respectively) to the enzymes from *Clostridium thermocellum* (PDB: 3qde) (Bianchetti et al., 2011) and *Cellvibrio gilvus* (PDB: 2cqs) (Hidaka et al., 2006). The protein derived from E9SAK5 aligned with the same sequences presenting much lower identity and similarity (30% and 45%, respectively). The alignment of E9SAK5 with members of the GH94 family showed higher sequence similarities with *Vibrio proteolyticus* ChBP, and *Vibrio furnissii* CBP, with an identity of 37% and a similarity of 68% for both proteins. Sequence alignments showed the conservation of critical amino acids involved in the phosphate-binding site and other residues conforming substrate-binding subsites −1, +1, and +2 (Figure 1).

**FIGURE 2:**
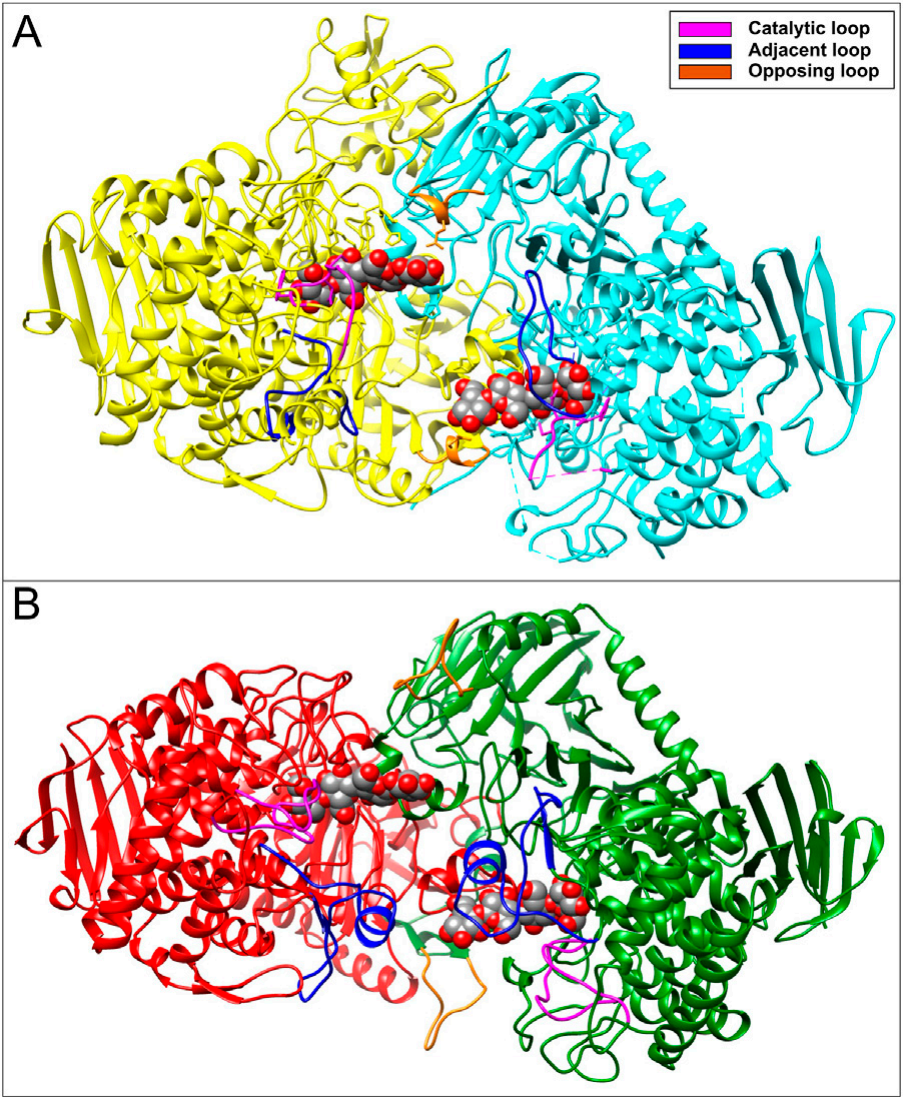
**(A)** Crystal structure of *Ct*CDP (PDB code: 5zn8) and **(B)** 3D-model of *Ral*CDP homodimer structure performed by Alphafold2. Both structures show the interaction with cellotetraose displayed as colored spheres.

Structure comparison performed between *V. proteolyticus* ChBP (PDB code: 1v7x) (Hidaka et al., 2004), *C. thermocellum* CBP (PDB code: 3qde) (Bianchetti et al., 2011), *Ct*CDP (PDB code: 5nz8) (O’Neill et al., 2017), and a model of the E9SAK5 protein designed by AlphaFold2 (Jumper et al., 2021) revealed significant structural differences in catalytic, adjacent, and opposite loops length (Figures 2A, B). Based on these dissimilarities, we assumed that the protein sequence from entry E9SAK5 corresponded to *R. albus* 8 cellodextrin phosphorylase (*Ral*CDP), despite its relatively low sequence similarity with other CDPs, like *Thermoclostridium stercorarium* (53%), and *Thermotoga neapolitana* (49%). In addition, multiple sequences analysis of several glycosyl phosphorylases from the family GH94 revealed that the N-terminal domain presents a highly conserved region (amino acids 1–63). This region was predicted to be involved in substrate binding, homodimer interaction, and to be a putative CBM by the SMART tool (Letunic and Bork, 2018; Letunic et al., 2021). As far as we know, no other glycosyl phosphorylase from this family was reported to possess a CBM.

### 3.2 Production of recombinant enzymes

**FIGURE 3:**
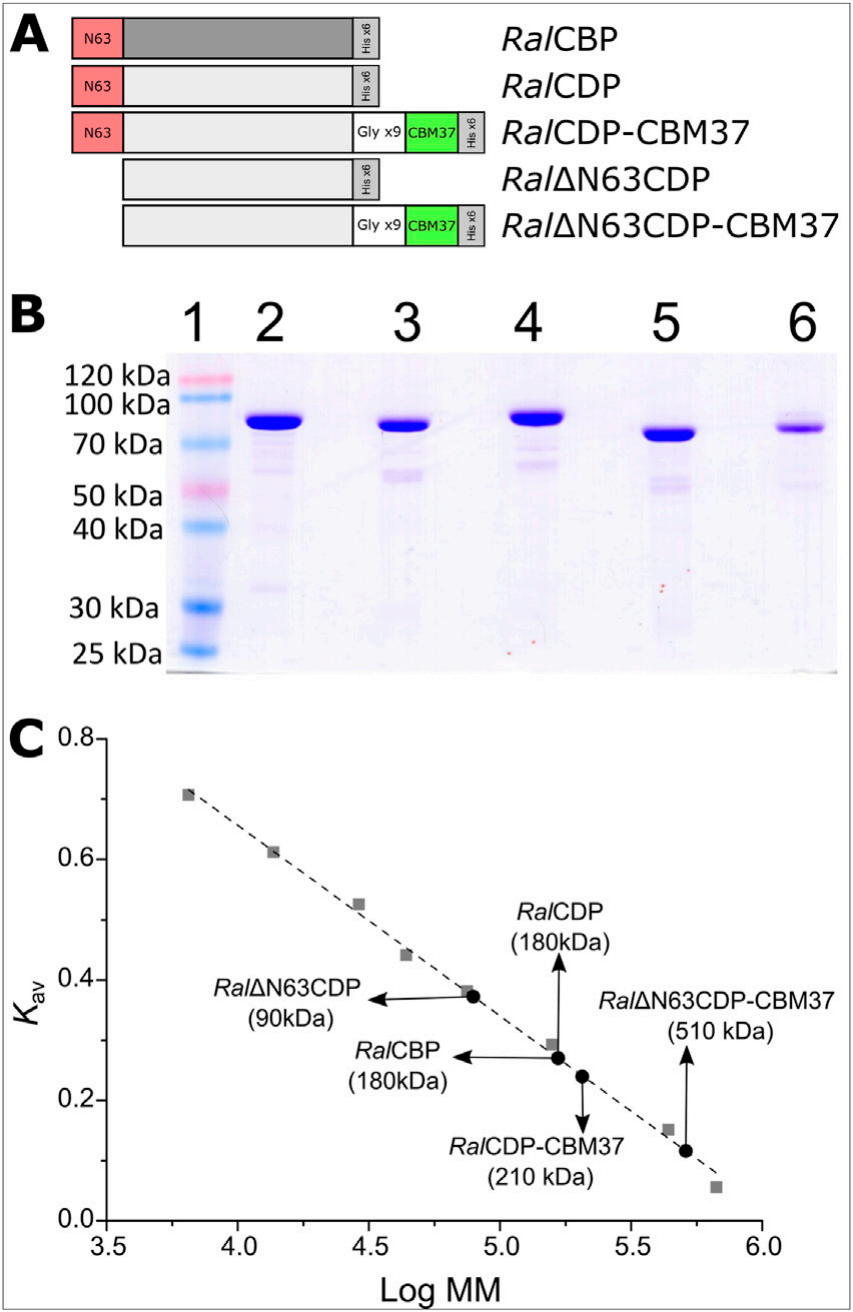
Schematic representation of CDP variants **(A)**. SDS-Page of purified proteins: Molecular mass marker (1), *Ral*CBP (2), *Ral*CDP (3), *Ral*CDP-CBM37 (4), *Ral*∆N63CDP (5) and *Ral*∆N63CDP-CBM37 (6) **(B)**. Quaternary structure determination for each enzyme variant by size exclusion chromatography **(C).**

*Ruminococcus albus 8* CBP and four variants of CDP were constructed and produced in *E. coli*, as described in Figure 3. Thus, the constructs were designed to produce *Ral*CBP (the E9SB92 protein), and *Ral*CDP (the E9SAK5 protein) and its truncated version lacking the firsts sixty-three N-terminal amino acids (Ral∆N63CDP). Also, we joined the CBM37 by a flexible glycine linker to *Ral*CDP and Ral∆N63CDP to construct the fusion proteins *Ral*CDP-CBM37 and Ral∆N63CDP-CBM37 (Figure 3A). Flexible linkers are the most often employed since they allow freedom of mobility and interaction between domains (Martins et al., 2020). The applied strategy enables fusing the CBM to the enzymes’ N- or the C-extreme. Still, all resulting enzymes were inactive when the CBM was linked to the N- terminal. As also illustrated in Figure 2A, all the recombinant proteins were produced with a His-tag fused to its C-terminal and purified by Ni^2+^-affinity (Ni^2+^-IDA) chromatography.

Figure 3B shows that recombinant proteins were produced and purified to a high electrophoretic degree. *Ral*CBP has 829 amino acids and a theoretical molecular mass of 92.3 kDa, while RalCDP has 809 amino acids and 90.2 kDa. Analysis by SDS-PAGE of the purified proteins exposed a band of approximately 90 kDa. Gel filtration chromatography on Superdex 200 indicated that both enzymes are homodimers with a molecular mass of 180 kDa (Figure 3C). These data are in good agreement with that reported for other enzymes of the GH94 family previously characterized (Kim et al., 2002; Krishnareddy et al., 2002; Nakai et al., 2010; Bianchetti et al., 2011). Meanwhile, the mutant Ral∆N63CDP eluted as a single peak with an elution volume corresponding to a monomer. This latter result suggests a loss of interaction between the subunits consequent with the absence of the conserved region on the N-terminal. Conversely, adding the CBM37 to the C-extreme of *Ral*CDP did not disturb the homodimer conformation. On the other hand, the CBM37 fusion to the C-terminal of Ral∆N63CDP produced a protein oligomer that eluted in a volume corresponding with a homo-pentamer (Figure 3C).

We measured the enzymatic activity of each elusion peak by assaying the respective enzyme in the synthesis sense of catalysis. *Ral*CBP and *Ral*CDP presented a specific activity of 57 U/mg and 98 U/mg, with glucose and cellobiose as substrates, respectively. For the truncated version Ral∆N63CDP, this value was 31 U/mg, despite its conformational change from a homodimer to a monomer. Different results were observed with the fusion of the CBM to these enzymes. *Ral*CDP-CBM37 showed a specific activity of 45 U/mg; meanwhile, Ral∆N63CDP-CBM37 was completely inactive, probably due to its oligomeric state. These results show the importance of the N-terminal extreme of *Ral*CDP, as the absence of such a region prevents the protein’s correct conformation as a homodimer, with a parallel decrease in enzyme activity. In addition, the mere presence of the His-tag or the CBM fusion at the N-terminal demolished the enzymatic activity.

### 3.3 Study of physicochemical properties

**FIGURE 4:**
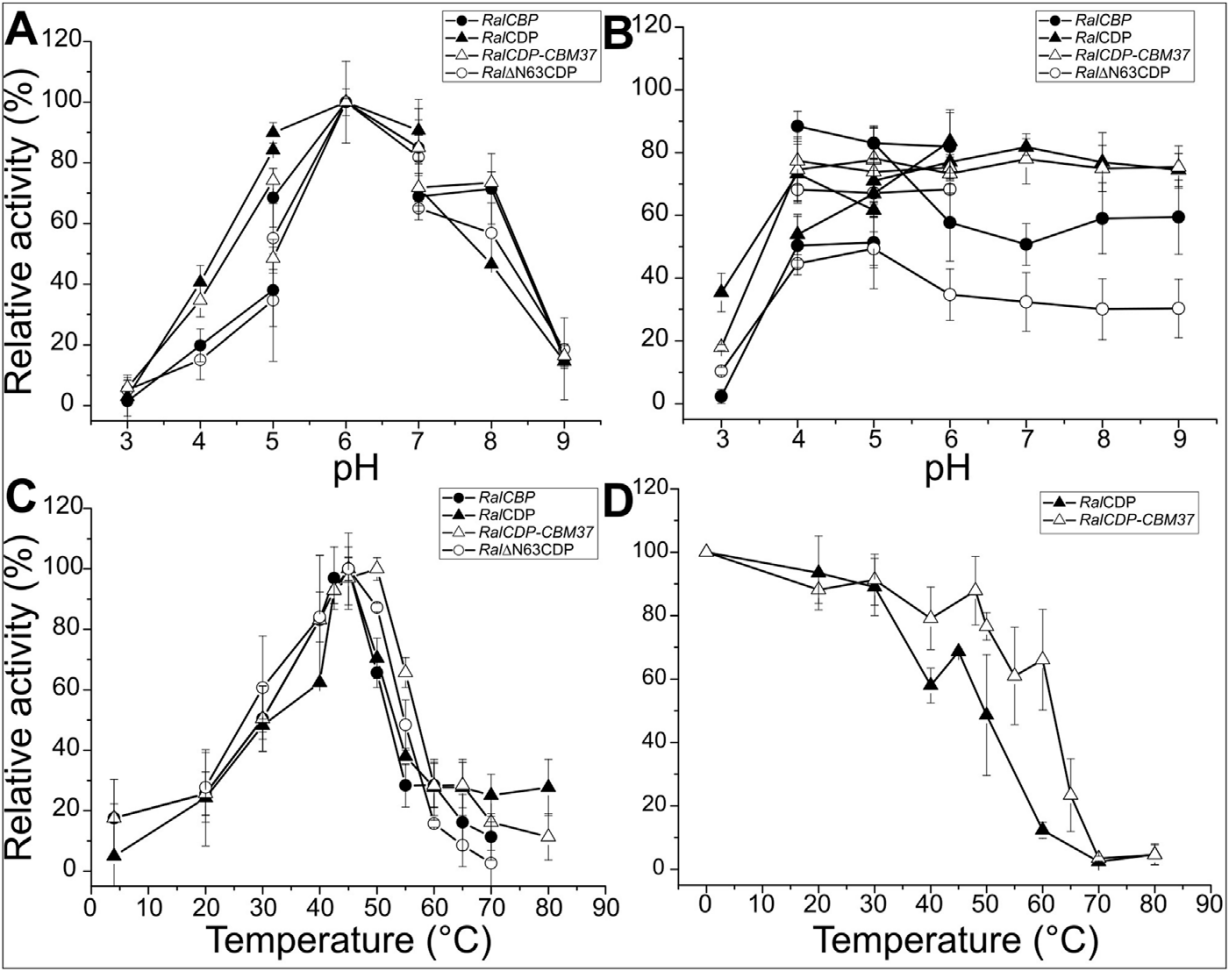
Enzymatic activity of *Ral*CBP (black circles), *Ral*CDP (black triangles), *Ral*CDP-CBM37 (white triangles) and *Ral*∆N63CDP (white circles) at different pHs **(A)**. Residual enzymatic activity of *Ral*CBP (black circles), *Ral*CDP (black triangles), *Ral*CDP-CBM37 (white triangles), and *Ral*∆N63CDP (white circles) after being incubated for 1 h in different buffer solutions **(B)**. Enzymatic activity of *Ral*CBP (black circles), *Ral*CDP (black triangles), *Ral*CDP-CBM37 (white triangles) and *Ral*∆N63CDP (white circles) at different temperatures**(C)**. Residual enzymatic activity of *Ral*CDP (black triangles), *Ral*CDP-CBM37 (white triangles) after 1 h incubations at different temperatures **(D)**.

We examined the activity of the recombinant enzymes under study at different pH and temperature ranges since the modifications made to their sequences could alter the protein’s sensitivity to pH or temperature. No optimum pH and temperature differences were evident between any enzyme’s phosphorolytic and synthetic activities. *Ral*CDP exhibited maximum activity at pH range 5–7, but the proteins *Ral*CBP, *Ral*∆N63CDP, and *Ral*CDP-CBM37 showed a narrower maximum activity peak at pH 6–7. At pH 5, *RalCBP* and *Ral*∆N63CDP displayed a reduction of 60% in their respective catalytic activity compared with *Ral*CDP and *Ral*CDP-CBM37. In the same way, a slight difference (∼20%) was detected for the mutant enzymes at pH 8.0 (Figure 4A). Regarding the proteins’ stability at different pH conditions, *Ral*CDP and *Ral*CDP-CBM37 conserved 80% of their enzymatic activity after incubating for 1 h in the pH range 4–9. *Ral*CBP only preserved this activity level when incubated in the pH range of 4–6. Meanwhile, in the rest of the conditions tested, *Ral*CBP showed around 50%–60% of its catalytic activity. *Ral*∆N63CDP was the enzyme with the least stability, with 40% residual activity after incubation with sodium citrate buffer (pH 4–5) or potassium phosphate (pH 5–9). However, this enzyme conserved 70% residual activity when incubated in sodium acetate buffer (between pH 4–6). Also, all enzymes showed shallow stability when incubated in sodium citrate buffer at pH 3.0 for 1 h (Figure 4B).

Determination of optimal temperature for catalytic activity showed that *Ral*CBP, *Ral*CDP, and *Ral*∆N63CDP presented a maximum at 45°C. However, *Ral*CDP activity decreased considerably at temperatures slightly lower or higher than its optimal temperature (60% and 40% of its optimal activity at 40°C and 55°C, respectively). It was noticed that the fusion of the CBM37 domain to *Ral*CDP improved the enzyme´s behavior toward temperature. Thus, *Ral*CDP-CBM37 presented a maximum activity at 50°C with a reduction to 80% and over 60% values at 40°C and 55°C, respectively (Figure 4C). To analyze the proteins’ thermal stability, both enzymes were incubated for 1 h at different temperatures to measure the residual enzymatic activity later. *Ral*CDP-CBM37 retained more than 60% of its catalytic capacity up to 60°C, while at this temperature, the *Ral*CDP activity reduced by 90% (Figure 4D). The global of protein stability was reinforced by experiments determining melting temperatures for these enzymes shown in Supplementary Figure S2.

### 3.4 Kinetic characterization of *Ruminococcus albus* CBP and CDP variants

We further characterized the recombinant phosphorylases determining their kinetic properties when using different substrates in both senses of catalysis, with results detailed in Table 1. We also analyzed if the CBM addition improves enzyme activity on long-chain oligosaccharides or if the quaternary structure change (due to the lack of the N-terminal region in the protein) could modify the active site conformation, opening it to broader substrates. We tested the phosphorolytic and synthetic activities of *Ral*CBP and the active CDP forms (RalCDP, Ral∆N63CDP, and RalCDP-CBM37) with substrates with different DP like glucose, cellobiose, cellotriose, cellotetraose, cellopentaose, and the two cellulosic oligosaccharides CMC and PASC.

**TABLE 1:** Kinetic parameters from the different enzyme variants with substrates with different DP. ^a^units for substrates CMC and PASC ND: Not detected activity even at a 50-fold increase in enzyme concentration.

Enzyme	Reaction	Substrate	*k*_cat_ (s^-1^)	*K*_M_ (mM) (mg/mL)
*Ral*CBP	Synthesis	Glucose	178 ± 2	2.0 ± 0.2
		Cellobiose	0.41 ± 0.01	9.0 ± 0.6
		Cellotriose	0.14 ± 0.01	9.4 ± 0.6
		Cellotetraose	ND	ND
		CMC	ND	ND
		PASC	ND	ND
	Phosphorolysis	Cellobiose	152 ± 4	2.3 ± 0.4
		Cellotriose	0.62 ± 0.01	5.2 ± 0.9
		Cellotetraose	ND	ND
		CMC	ND	ND
		PASC	ND	ND
*Ral*CDP	Synthesis	Glucose	4.98 ± 0.08	0.8 ± 0.1
		Cellobiose	310 ± 30	8.0 ± 1
		Cellotriose	720 ± 30	13 ± 3
		Cellotetraose	1416 ± 38	9 ± 3
		Cellopentaose	235 ± 9	1.5 ± 0.4
		CMC	0.33 ± 0.03	6.5 ± 0.5
		PASC	0.38 ± 0.03	8.4 ± 0.1
	Phosphorolysis	Cellobiose	19.7 ± 0.4	4.3 ± 0.4
		Cellotriose	29.6 ± 0.5	1.9 ± 0.4
		Cellotetraose	28.2 ± 0.5	0.28 ± 0.08
		Cellopentaose	20.4 ± 0.3	0.13 ± 0.03
		CMC	0.024 ± 0.001	5.5 ± 0.4
		PASC	0.028 ± 0.001	5.1 ± 0.8
*Ral*CDP-CBM37	Synthesis	Glucose	ND	ND
		Cellobiose	149 ± 9	5.8 ± 0.8
		Cellotriose	757 ± 15	11 ± 2
		Cellotetraose	1474 ± 28	8 ± 1
		Cellopentaose	218 ± 6	1.3 ± 0.3
		CMC	0.27 ± 0.03	3.0 ± 0.5
		PASC	0.27 ± 0.03	2.1 ± 0.4
	Phosphorolysis	Cellobiose	1.2 ± 0.1	11 ± 2
		Cellotriose	30.2 ± 0.4	2.2 ± 0.3
		Cellotetraose	27.5 ± 0.4	0.24 ± 0.06
		Cellopentaose	16.4 ± 0.2	0.14 ± 0.03
		CMC	0.28 ± 0.04	2.8 ± 0.3
		PASC	0.27 ± 0.03	3.6 ± 0.7
*Ral*∆N63CDP	Synthesis	Glucose	0.47 ± 0.4	11 ± 2
		Cellobiose	42 ± 5	22 ± 2
		Cellotriose	0.02 ± 0.01	7 ± 2
		Cellotetraose	ND	ND
		CMC	ND	ND
		PASC	ND	ND
	Phosphorolysis	Cellobiose	0.02 ± 0.01	20 ± 3
		Cellotriose	ND	ND
		Cellotetraose	ND	ND
		CMC	ND	ND
		PASC	ND	ND

**FIGURE 5:**
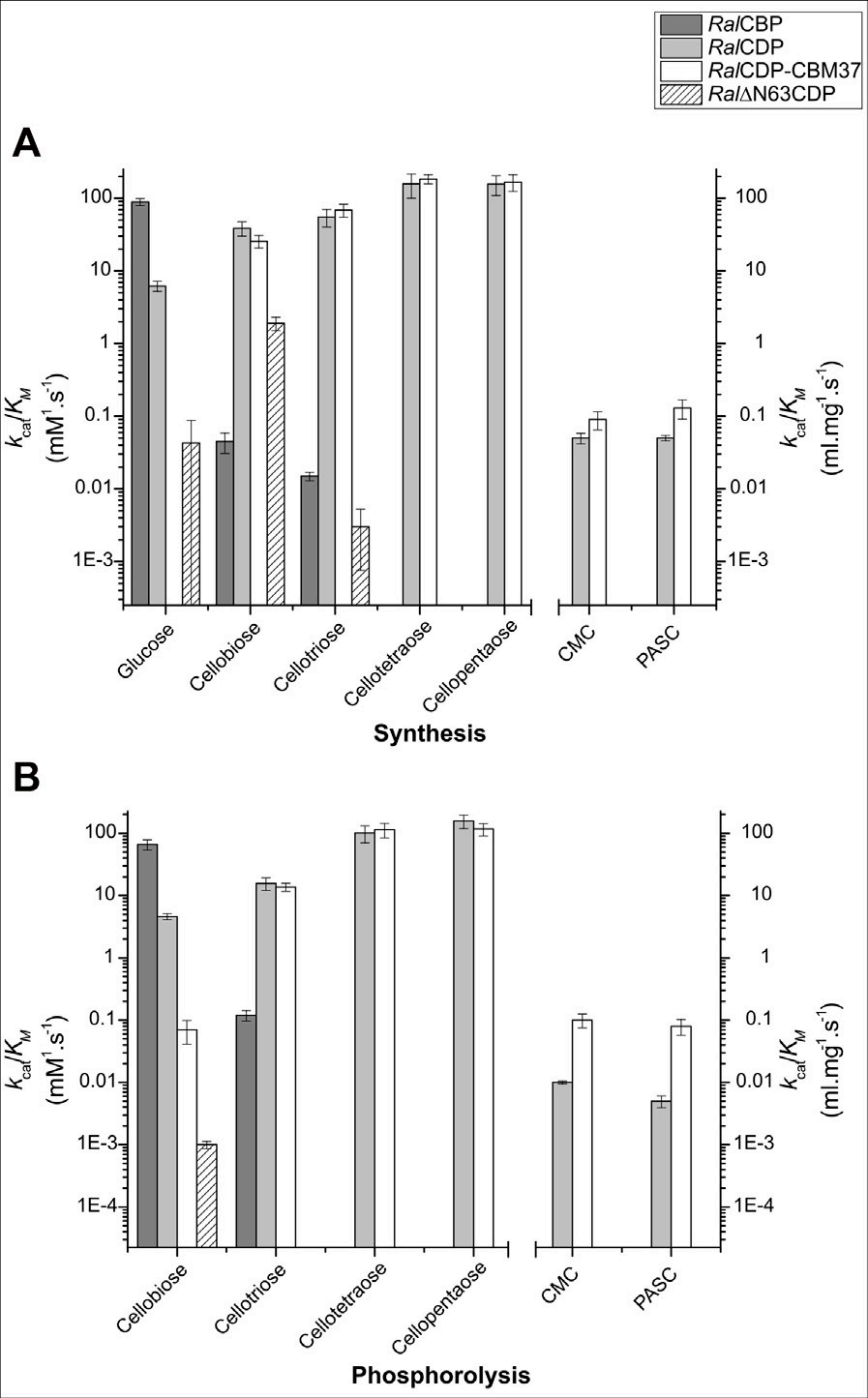
Enzymatic efficiencies of *Ral*CBP (dark gray), *Ral*CDP (light gray), *Ral*CDP-CBM37 (white), and *Ral*∆N63CDP (striped) with cello-oligosaccharides of different DP for synthesis **(A)** and phosphorolysis **(B)**.

Table 1 shows that *Ral*CBP exhibited phosphorolytic activity only with cellobiose and cellotriose. The kinetic parameters indicated a higher (by more than two orders of magnitude) catalytic performance of the enzyme with cellobiose (Figure 5). Indeed, the major difference was that the *k*_cat_ value with cellotriose was 250-fold lower than that obtained with the disaccharide, although both *K*_M_ values were similar (only 2-fold lower for cellobiose) (Table 1). Concerning synthetic activity using Glc-1P as the donor substrate, *Ral*CBP exhibited maximum catalytic efficiency with glucose as the acceptor (Figure 5). The DP of the acceptor substrate affected considerably *Ral*CBP catalytic ability. Compared with glucose, decreases of 450- and 1,100-fold in *k*_cat_ were determined with cellobiose and cellotriose, respectively, but *K*_M_ values exhibited increases of ∼5-fold (Table 1). Overall, the kinetic behavior of *Ral*CBP agrees with its identification of cellobiose phosphorylase.

Data obtained in the RalCDP characterization as a cellodextrin phosphorylase were markedly different from the above described for RalCBP. Thus, RalCDP showed synthetic activity with multiple acceptors, like glucose, cellobiose, cellotriose, cellotetraose, cellopentaose, and, less efficiently, the polysaccharides PASC and CMC (Table 1). The catalytic efficiency with the monosaccharide was considerably lower than with the other soluble substrates or *Ral*CBP (Figure 5). Indeed, RalCDP exhibited maximum catalytic efficiency with cellotetraose and cellopentaose substrates and values 3-, 4- and 25-fold lower for cellotriose, cellobiose and glucose, respectively. On the other hand, the enzyme’s catalytic capacity for using the polysaccharides CMC and PASC was significantly low (Table 1; Figure 5). These results indicate the preference of this enzyme for oligosaccharides over the use of di- or monosaccharides as acceptors. However, the activity decreased when the DP increased considerably, like CMC and PASC polysaccharides, as described for other CDPs [54]. Similarly, *Ral*CDP achieved maximum catalytic efficiencies in the phosphorolysis direction with cellopentaose and cellotetraose, diminishing in one order of magnitude by cellotriose and cellobiose (Figure 5). This decrease in catalytic efficiency was due to increased K_M_ values as it decreased the substrate’s polymerization degree. In contrast, the *k*_cat_ values were similar with all cello-oligosaccharides. Otherwise, phosphorolytic activity (in terms of *k*_cat_) with polysaccharides CMC and PASC was 1,000-fold lower (Table 1).

The N-terminal region removal provided notably different kinetic parameters to Ral∆N63CDP compared with the wild-type RalCDP. As shown in Table 1, the truncated enzyme exhibited a ∼1,000-fold reduction in its phosphorolytic capacity with cellobiose and was inactive with other disaccharides or oligosaccharides. Besides, the analysis of the synthetic reaction showed a 10-fold lower kcat for the elongation of cellobiose with Glc-1P. Other differences in kinetic behavior observed between Ral∆N63CDP and RalCDP for catalysis in the synthesis and phosphorolysis directions refer to affinity toward the substrates Glc-1P and phosphate (Table 2). Ral∆N63CDP showed a ∼30-fold reduction in phosphate affinity, leading to a significant decrease in its catalytic efficiency for phosphorolysis. However, the capacity for catalyzing the synthetic reaction with cellobiose remains at the same level as the wild-type RalCDP.

**TABLE 2:** Kinetic parameter for Glc-1P and Pi from the different enzyme variants.

Enzyme	Substrate	Synthesis			Phosphorolysis		
		*k*_cat_ (s^-1^)	*K*_M_ Glc-1P (mM)	*k*_cat_/*K*_M_ Glc-1P	*k*_cat_ (s^-1^)	*K*_M_ Pi (mM)	*k*_cat_/*K*_M_ Pi (s^-1^.mM^-1^)
*Ral*CBP	Glucose	178 ± 2	2.0 ± 0.2	89	-	-	-
	Cellobiose	0.41 ± 0.01	15 ± 3	0.027	284 ± 1	0.58 ± 0.02	489.6
*Ral*CDP	Glucose	5.0 ± 0.1	1.4 ± 0.9	3.6	-	-	-
	Cellobiose	310 ± 24	2.1 ± 0.1	147.6	19.7 ± 0.4	0.5 ± 0.1	39.4
*Ral*∆N63CDP	Glucose	0.47 ± 0.04	0.14 ± 0.08	3.4	-	-	-
	Cellobiose	42 ± 5	0.32 ± 0.08	131.3	0.02 ± 0.01	16 ± 5	0.001

On the other hand, the fusion of the CBM37 to Ral∆N63CDP and *Ral*CDP had quite varied effects on the catalytic properties. Ral∆N63CDP-CBM37 was completely inactive, while *Ral*CDP-CBM37 showed similar kinetic parameters to *Ral*CDP when using soluble oligosaccharides as substrates (Table 1). However, for the polysaccharides CMC and PASC, the CBM-tagged protein enhanced the *k*_cat_ for phosphorolysis by one order of magnitude. It exhibited a 2-fold reduction in *K*_M_ for CMC and PASC for both reaction directions (Table 2). This increased enzyme affinity for polysaccharides substrates enhances by ∼20-fold the catalytic efficiency for phosphorolysis compared to *Ral*CDP (Figure 5).

### 3.5 Carbohydrate binding assay

**FIGURE 6:**
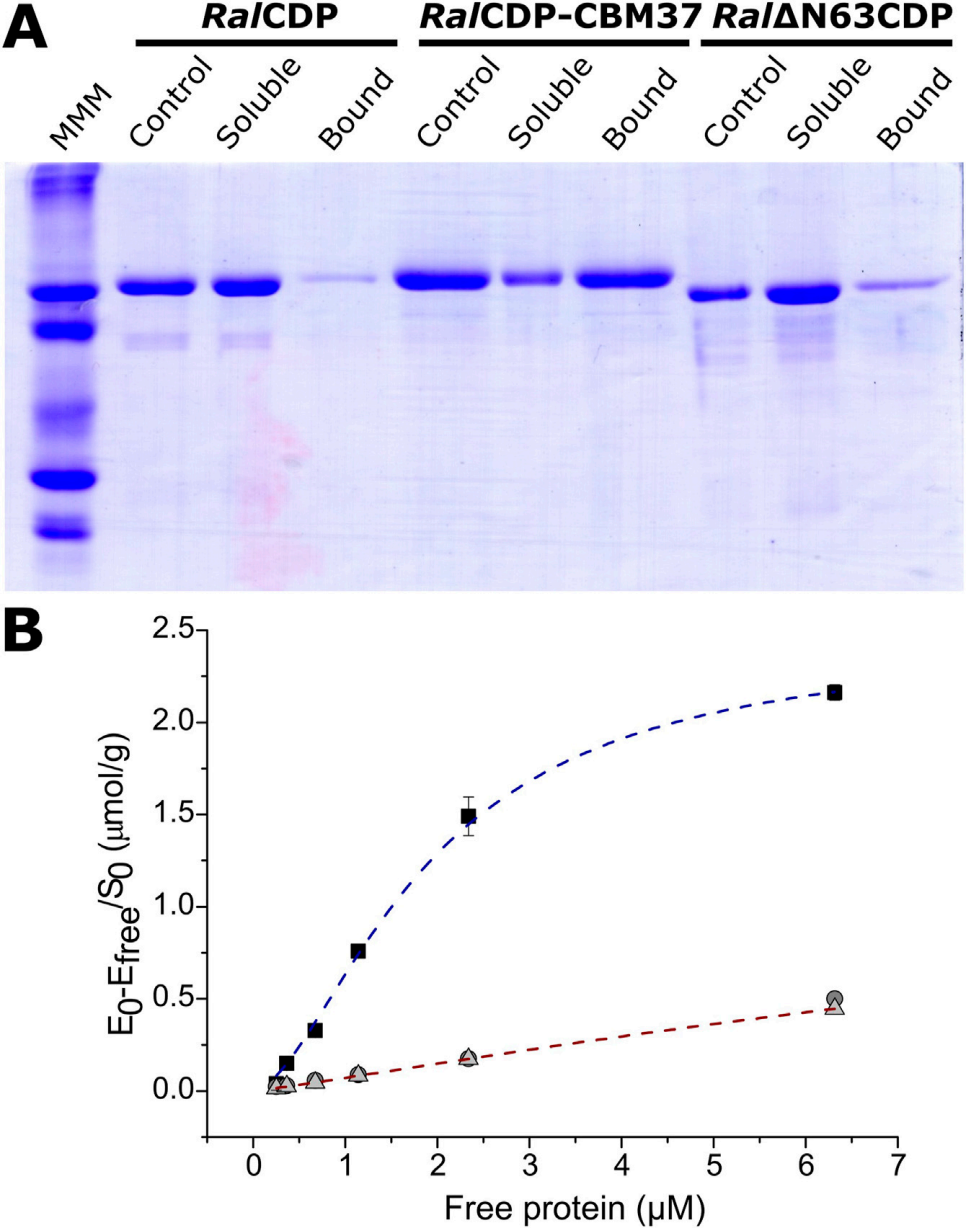
Analysis of CDP variants binding capacity to PASC. SDS-PAGE of the different fractions after incubation with the substrate (Control: enzyme incubated without substrate) **(A)**. Binding isotherms for *Ral*CDP (circles), *Ral*CDP-CBM37 (squares), and *Ral*∆N63CDP (triangles) **(B)**.

The binding capacity of *Ral*CDP, *Ral*∆N63CDP, and *Ral*CDP-CBM37 to insoluble cellulosic polysaccharides was studied by incubating the enzymes with PASC. After incubation, the remaining soluble and bound fractions were analyzed by SDS-PAGE. As shown in Figure 6A, *Ral*CDP could not bind to the insoluble polysaccharide PASC, remaining mainly in the soluble fraction after incubation. The same result was obtained for *Ral*ΔN63CDP, indicating that the N-terminal region, predicted as CBM, lacks a carbohydrate-binding function, at least to cellulosic substrates. However, the addition of CBM37, exemplified by *Ral*CDP-CBM37, remarkably improved its binding capacity to this polysaccharide. The increment supports this result in enzyme affinity to polysaccharides observed in the kinetics parameters determined for this enzyme.

We calculated the respective binding parameters from the isotherm of the interaction betweenRalCDP-CBM37 and PASC, shown in Figure 5B. The fusion protein presented a dissociation constant (*K*_d_) of 1.73 ± 0.06 µM and an adsorption capacity (*A*_max_) of 2.36 ± 0.05 μmol/g. This adsorption capacity is 5-fold higher than that of enzymes lacking a CBM. These results indicate that CBM37 effectively recognizes the insoluble substrate PASC, also reinforcing the absence of functionality of the protein’s N-terminal domain for binding to the polysaccharide, as supported by results obtained with the complete *Ral*CDP (Figure 6A). The increment in substrate binding affinity to the insoluble substrate was also reflected in the kinetic parameters of *Ral*CDP-CBM37, which presents a decrease in *K*_M_ with PASC compared to *Ral*CDP (Table 1).

### 3.6 Evaluation of polysaccharides synthesis and degradation

**FIGURE 7:**
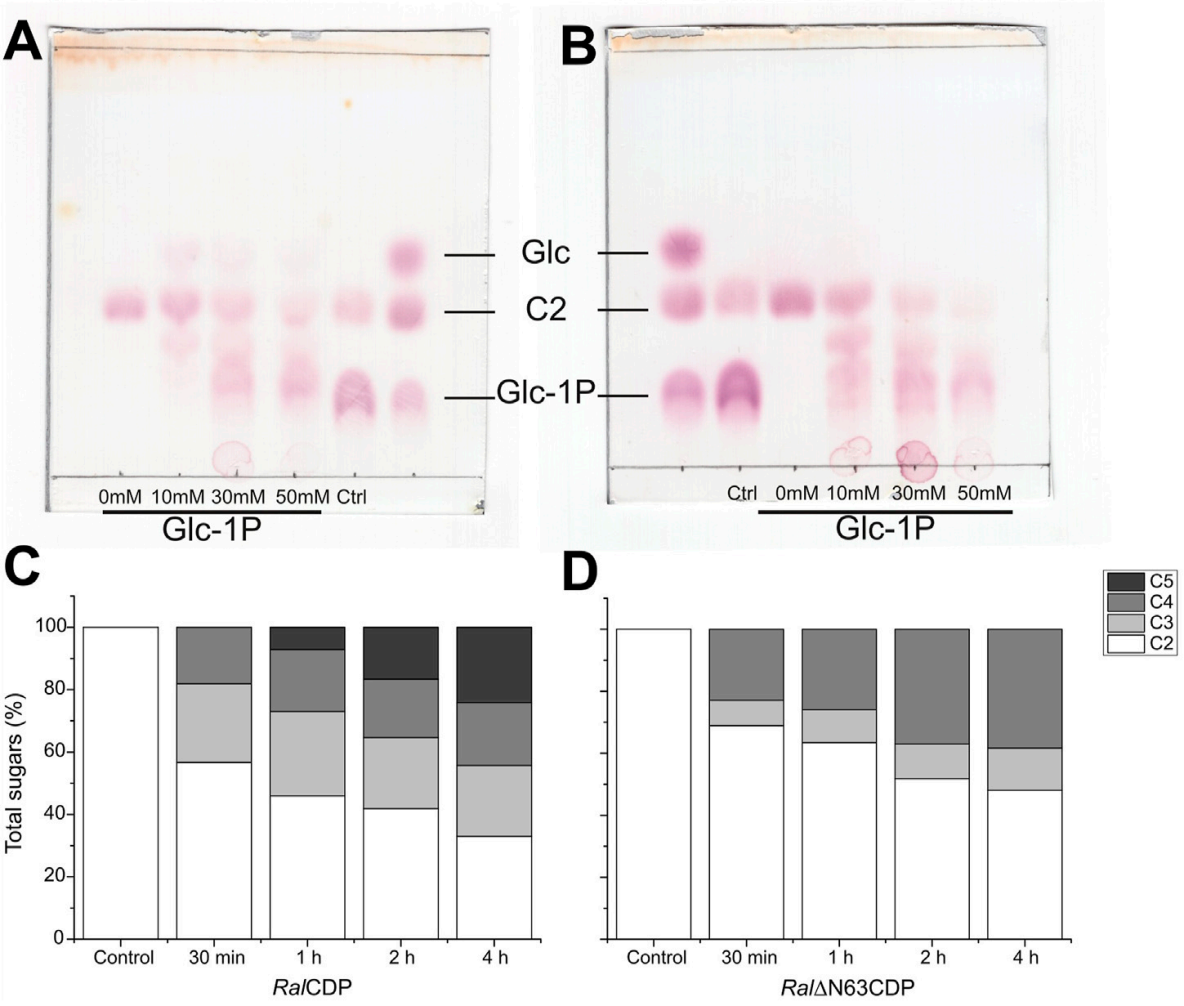
TLC analysis of *Ral*CDP **(A)** and *Ral*ΔN63CDP **(B)** reactions at different Glc-1P: cellobiose ratios. HPLC analysis of cello-oligosaccharides synthesized by *Ral*CDP **(C)** and *Ral*ΔN63CDP **(D)**.

The synthesis of polysaccharides by *Ral*CDP and *Ral*ΔN63CDP was monitored utilizing cellobiose (10 mM) and different concentrations of Glc-1P (10, 30, and 50 mM) as substrates. Reaction products were analyzed by TLC (Figures 7A, B). In the *Ral*CDP reactions, glucose can be observed as a result of the phosphorolytic activity of this enzyme, mainly at low Glc-1P concentrations (Figure 7A). However, this did not occur in reactions catalyzed by RalΔN63CDP for any of the Glc-1P concentrations tested (Figure 7B). The absence of glucose in RalΔN63CDP reactions is a consequence of its low affinity for Pi, as was shown in its kinetic characterization (Table 2). In addition, at low Glc-1P concentration (10 mM), we observed the synthesis of higher DP saccharides with the catalysis of RalΔN63CDP than that exerted by RalCDP, reflecting reaction displacement towards polysaccharides synthesis.

The use of HPLC allowed further analysis of the reaction products of both enzymes. The reactions were performed from 30 min to 4 h using Glc-1P and cellobiose in a 3:1 ratio. As illustrated by Figure 7C, *Ral*CDP produced cellotriose, cellotetraose (∼25 and ∼20%, respectively), and increasing amounts of cellopentaose over the reaction time course (7, 17, and 24% for 1, 2, and 4 h, respectively). Meanwhile, in the reactions catalyzed by *Ral*∆N63CDP, only cellotriose and cellotetraose were produced (Figure 7D). The percentage of cellotriose detected was lower than those from *Ral*CDP reactions. However, increasing amounts of cellotetraose were produced over time (23%–38%), being higher than those from *Ral*CDP (18%–20%). These differences in reaction products could be attributed to the fact that *Ral*∆N63CDP cannot extend the oligosaccharide chain beyond cellotetraose, as shown in its kinetic characterization (Table 1), giving rise to the accumulation of this oligosaccharide. While, *Ral*CDP has a high enzymatic activity with cellotetraose and cellopentaose. For these reasons, the *Ral*CDP reaction would channel cellotetraose toward synthesizing oligosaccharides with a higher DP.

**FIGURE 8:**
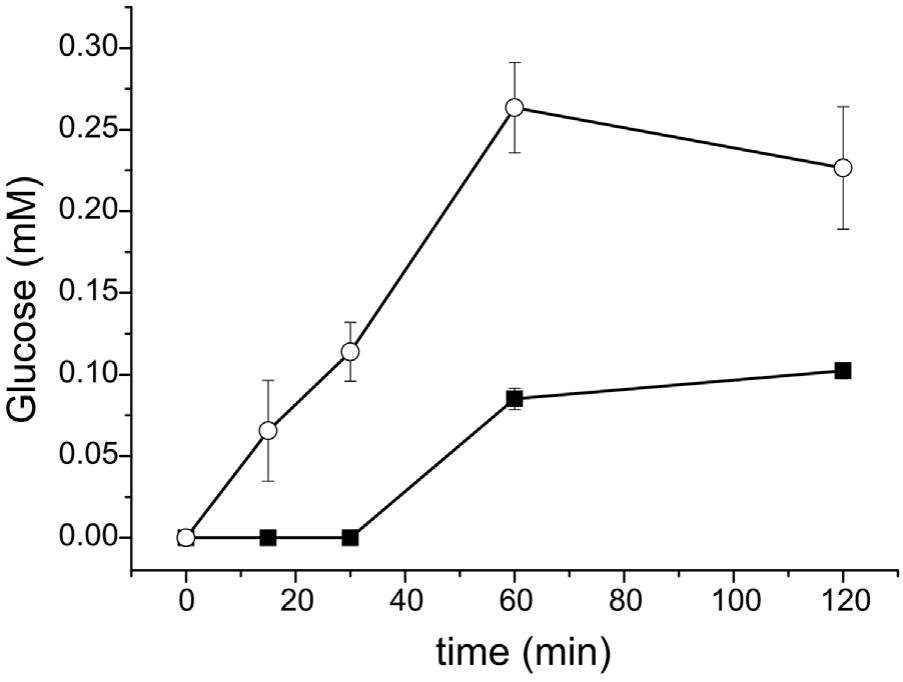
Glucose released from PASC arsenolysis with *Ral*CDP (squares) and *Ral*CDP-CBM37 (circles).

The higher catalytic efficiency of *Ral*CDP-CBM37 with cellulosic polysaccharides could be an advantage for its application in different processes. Its higher phosphorolytic capacity could be useful for the production of Glc-1P from high available polysaccharides like cellulose. Thus, we followed the arsenolysis of PASC at different reaction times catalyzed by *Ral*CDP and *Ral*CDP-CBM37, the two enzyme versions exhibiting higher phosphorolytic activity. The strategy of replacing phosphate with arsenate was previously useful for measuring phosphorolytic activity on the insoluble polysaccharide PASC (Eis and Nidetzky, 1999; Zhang and Lynd, 2005). Figure 8 shows that RalCDP-CBM37 presented a ∼4-fold higher activity than RalCDP (0.71 vs. 0.16 µmol glucose/µmol enzyme. min). A maximum glucose yield of 0.26 mM was obtained after a 1 h reaction with RalCDP-CBM37, while RalCDP presented a maximum glucose yield of 0.1 mM after 2 h. This higher rate of phosphorolysis seems to be caused by the enhanced adsorption capacity, increasing the effective local enzyme concentration on the insoluble substrate due to the addition of the CBM.

## 4 Discussion

CDPs are carbohydrate-active enzymes with outstanding potential for the biocatalytic bottom-up synthesis of β-glucans as major bioactive compounds. The donor substrate α-Glc-1P serves as a cheaper version of the UDP-Glc required by the corresponding “Leloir glycosyltransferases” and can be conveniently prepared through the phosphorolysis of cheap and abundant resources like starch or cellulose (Ubiparip et al., 2021). They have the broadest acceptor and donor substrate specificity of all GH94 enzymes, making them good tools for suitable biocatalytic production of diverse carbohydrates and related molecules. However, only a few CDPs were biochemically characterized (Sawano et al., 2013; O’Neill et al., 2017; Wu et al., 2017; Ubiparip et al., 2021).

In this work, we identify a GH94 glycosyl phosphorylase from *R. albus* that showed relatively low sequence similarity with other CDPs, like *T. stercorarium* (53%), and *T. neapolitana* (49%). In addition, structure comparison with members of the GH94 family like *V. proteolyticus* ChBP (PDB code: 1v7x) (Hidaka et al., 2004), *C. thermocellum* CBP (PDB code: 3qde) (Bianchetti et al., 2011) and *Ct*CDP (PDB code: 5nz8) (O’Neill et al., 2017), revealed significant sequence and structural differences in catalytic, adjacent, and opposite loops, which are highly involved in active site conformation. (O’Neill et al., 2017; Gabrielli et al., 2021). Here, we noticed that these differences led to an increase in the active site accessibility for long-chain oligosaccharides in *Ral*CDP, as shown in Figure 2.

As was hypothesized from structural analysis and confirmed by 3D-models in Figure 2, *Ral*CDP showed remarkable kinetic differences from other CDPs (detailed in Supplementary Table S2). This enzyme was active with substrates of different DP, ranging from monosaccharides to long-chain cellulosic oligosaccharides. Previously, *Ta*CDP/CBP showed activity on both monosaccharides and cello-oligosaccharides (Wu et al., 2017), but only *Ct*CDP showed activity on long-chain oligosaccharides (Ye et al., 2011). *Ral*CDP activity with monosaccharides was similar to that of *Ta*CDP/CBP but showed a 3- and 9-fold higher activity than *Ct*CDP, for PASC phosphorolysis and synthesis, respectively (Supplementary Table S2).

Despite CDPs being active on cello-oligosaccharides, they are single modular proteins with no apparent CBM. However, we noticed that *Ral*CDP presents a highly conserved N-terminal region, which is predicted to be involved in substrate binding, homodimer interaction, and/or a putative CBM. To analyze in depth the properties of these GH94 family proteins, we constructed two variants of *Ral*CDP, one truncated mutant (RalΔN63CDP) lacking the conserved N-terminal region and another one (*Ral*CDP-CBM37) fused to a CBM of family 37, unique from *R. albus* (Xu et al., 2004; Ezer et al., 2008). The exact mechanism of the CBM37 in the *R. albus* cellulase system is still unknown, but its members recognize and bind strongly to cellulose and numerous other polysaccharides (Xu et al., 2004).

Characterization of the recombinant proteins reveals that the conserved N-terminal region is critical to homodimer assembly since the construction on the truncated mutant RalΔN63CDP lead to a monomeric enzyme. This was also predicted in crystal structures of other GH94 enzymes (Hidaka et al., 2004; 2006; Bianchetti et al., 2011; Kuhaudomlarp et al., 2019). In addition, we identify that this modification of the quaternary structure decreased the enzyme capacity to catalyze the phosphorolysis of cellodextrins. Although, the activity in the direction of synthesis was only slightly affected in the truncated mutant. The kinetic analysis proved that differences in the enzymatic activity of monomeric RalΔN63CDP resulted from its affinity decrease toward phosphate and increase for Glc-1P. Kinetics parameters for Glc-1P and Pi of RalCDP showed that enzyme relative affinity for these substrates was 80- and 360-fold higher than those reported for *R. albus* NE1 CDP (Sawano et al., 2013) (Supplementary Table S2). These results indicate that the minimal differences observed in the sequence of the loops conforming to the active site lead to a significant difference in substrate recognition.

The reduced phosphorolytic capacity observed inRalΔN63CDP has been exploited for the synthesis of soluble cello-oligosaccharides from cellobiose and Glc-1P at lower donor: acceptor ratios than those reported from other CDPs wild-type versions (Zhong et al., 2019; Zhong and Nidetzky, 2020; Nidetzky and Zhong, 2021). RalΔN63CDP produced increasing amounts of cellotetraose over the reaction time course (Supplementary Figure S3). This confirms that this enzyme cannot extend the polysaccharide chain over four glucose units, as previously noticed in its kinetic characterization. These features of *Ral*ΔN63CDP make it an excellent molecular tool for synthesizing short-chain cello-oligosaccharides (Ubiparip, 2020; Zhong et al., 2020). Such soluble cellodextrins were proven to be useful dietary fibers and prebiotics that stimulate the growth of many healthy human gut bacteria more efficiently than inulin, trans-galacto-oligosaccharides, and cellobiose alone (Zhong et al., 2020). In addition, producing such controlled product composition eliminates the need for subsequent organic solvent purification steps.

Worthy, CDPs could also be employed to degrade β-glucans by phosphorolysis and produce high-value Glc-1P from a cheap and easily accessible substrate like cellulose. Kinetic characterization of *Ral*CDP showed higher catalytic efficiency for the synthetic rather than the phosphorolytic reaction with cellulosic polysaccharides PASC and CMC. However, the fusion of the CBM37 to the C-terminal of *Ral*CDP improved both the *k*_cat_ for phosphorolysis and the enzyme affinity to insoluble cellulosic substrates (without affecting homodimer assembly). The increase in phosphorolytic activity here achieved by the fusion of the CBM37 to *Ral*CDP was 13.5-fold higher than that from the fusion of CBM9 to *Ct*CDP (Ye et al., 2011) (Supplementary Table S3). Thus, *Ral*CDP-CBM37 would constitutes an improved molecular tool to degrade cellulosic polysaccharides generating Glc-1P. Also, the fusion of CBM37 increased the synthetic reaction’s catalytic efficiency, mainly reducing the enzyme *K*_M_ (Supplementary Table S2). These effects on *K*_M_ and catalysis with insoluble substrates were reported in studies fusing CBMs to different single-domain enzymes like cellulases, xylanases, or laccases. (Ravalason et al., 2009; Zhang et al., 2013; Dai et al., 2021).

The analysis of the CDPs binding capacity suggests that *Ral*CDP and RalΔN63CDP could not bind to insoluble polysaccharides (PASC). However, the fusion of the CBM37 to *Ral*CDP enhanced its adsorption capacity by 5-fold. The adsorption parameters obtained for this construct were similar to those achieved by the fusion of CBM9 to *C. thermocellum* CDP (Ye et al., 2011) (Supplementary Table S4). In the same way, CBM9 fusion did not modify the kinetic parameters with soluble substrates but improved enzyme activity on insoluble cellulose. Despite the poorly understood CBM37 binding mechanism to cellulose, our results suggest that this domain may direct the enzyme towards cellulose chain ends increasing the *Ral*CDP-CBM37 activity and binding capacity to insoluble polysaccharides in the same level. Furthermore, a slight increment in optimum temperature and thermal stability was also observed after fusing CBM37 to *Ral*CDP (Figure 4D; Supplementary Figure S2). This stabilization effect was previously noticed by the fusion of CBM3 and CBM9 to GH5 cellulase (Maharjan et al., 2018) and by linking CBM72 to Umcel9A (Duan et al., 2017). In the same way, the removal of the CBM from the thermostable endoglucanase from Phialophora sp. G5 observed a decrease in the enzyme’s thermal stability (Zhao et al., 2012).

As a concluding remark, we proved that *Ral*CDP has activity with a wide range of substrates, from monosaccharides to long-chain polysaccharides. Also, we achieved two *Ral*CDP versions with valuable features. Ral∆N63CDP catalyzes the reaction with higher efficiency in the synthetic direction under low Glc-1P:cellobiose ratios, an advantageous characteristic for the tightly controlled synthesis of short-chain cello-oligosaccharides. Conversely, RalCDP-CBM37 has enhanced activity on polysaccharides, thus enabling the prospect of producing sugars-1P from highly available cellulosic substrates or synthesizing long-chain oligosaccharides.

## Data Availability

The datasets presented in this study can be found in online repositories. The names of the repository/repositories and accession number(s) can be found below: https://www.ncbi.nlm.nih.gov/nuccore/OP866768.1/, OP866768; https://www.ncbi.nlm.nih.gov/nuccore/OP866769.1/, OP866769.
