# Supplementary material for: Insights to improve the activity of glycosyl phosphorylases from Ruminococcus albus 8 with cello-oligosaccharides
Source: Front Chem. 2023 Apr 7;11:1176537. doi: 10.3389/fchem.2023.1176537 (PMC10119399; doi:10.3389/fchem.2023.1176537)
Supplement: Supplementary file 1 [file DataSheet2.PDF]

Supplementary Material

Insights to improve the activity of glycosyl phosphorylases from *Ruminococcus albus* 8 with cello oligosaccharides.

Alem Storani [1], Sergio A. Guerrero [1], Alberto A Iglesias *[1]

*** Correspondence:** Alberto A Iglesias: email: iglesias@fbcb.unl.edu.ar

Supplementary Figures and Tables

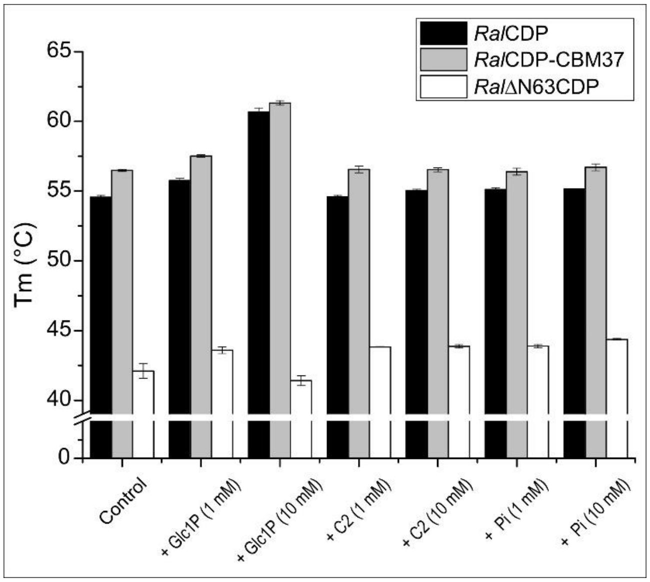

Supplementary Figure 2. Melting temperature comparison between *Ral*CDP variants in presence of different substrates. A real-time PCR device (StepOne PLUS Real Time PCR system) was used to monitor protein unfolding by the increase in the fluorescence of the fluorophor SYPRO Orange (Invitrogen, Carlsbad, CA). Protein samples (0.15 µg/µL) in 50 mM sodium acetate *buffer* (pH 6.0) containing the appropriate concentration of ligand (1-10 mM) in a reaction volume of 20 µL were incubated in 96-well microplates (MJ Research, Cambridge, MA) in the RT-PCR device. The samples were heated at 1°C per min, from 25°C to 95°C. The fluorescence emission intensity was measured by the device at 570 nm. Fluorescence intensities were plotted as a function of temperature by StepOne PLUS Real Time PCR system software. Tm was easily identified by plotting the first derivative of the fluorescence emission as a function of temperature (−dF/dT). Here, Tm is represented as the lowest part of the curve.
