# Supplementary material for: Insights to improve the activity of glycosyl phosphorylases from Ruminococcus albus 8 with cello-oligosaccharides
Source: Front Chem. 2023 Apr 7;11:1176537. doi: 10.3389/fchem.2023.1176537 (PMC10119399; doi:10.3389/fchem.2023.1176537)
Supplement: Supplementary file 2 [file Table1.DOCX]

Supplementary Material

Insights to improve the activity of glycosyl phosphorylases

from *Ruminococcus albus* 8 with cello oligosaccharides.

**Alem Storani ^1^, Sergio A. Guerrero ^1^, Alberto A Iglesias *^1^**

*** Correspondence:** Alberto A Iglesias: email: [iglesias@fbcb.unl.edu.ar](mailto:iglesias@fbcb.unl.edu.ar)

# Supplementary Figures and Tables

| Primer | Sequence (5´🡪3´) | Restriction sites |
| --- | --- | --- |
| FowGHCDP | GAGCTCCATCGGCCTGCCGGGTCGTTACG | *Sac*I |
| RevGHCDP | GTCGACGAAGCTTTTACTCGAGACCCATAACAACGG | *Sal*I- *Hind*III-*Xho*I |
| FowCBM37 | GGATCCGTACCCGACCAACGTGCAGG | *Bam*HI |
| RevCBM37 | GCGGCCGCGGTCAGGTTTTTCGGGG | *Not*I |

**Supplemental Table 1.** Primers used in this work
