# Supplementary material for: Insights to improve the activity of glycosyl phosphorylases from Ruminococcus albus 8 with cello-oligosaccharides
Source: Front Chem. 2023 Apr 7;11:1176537. doi: 10.3389/fchem.2023.1176537 (PMC10119399; doi:10.3389/fchem.2023.1176537)
Supplement: Supplementary file 3 [file DataSheet3.PDF]

Supplementary Material

Insights to improve the activity of glycosyl phosphorylases from *Ruminococcus albus* 8 with cello oligosaccharides.

Alem Storani [1], Sergio A. Guerrero [1], Alberto A Iglesias *[1]

*** Correspondence:** Alberto A Iglesias: email: iglesias@fbcb.unl.edu.ar

Supplementary Figures and Tables

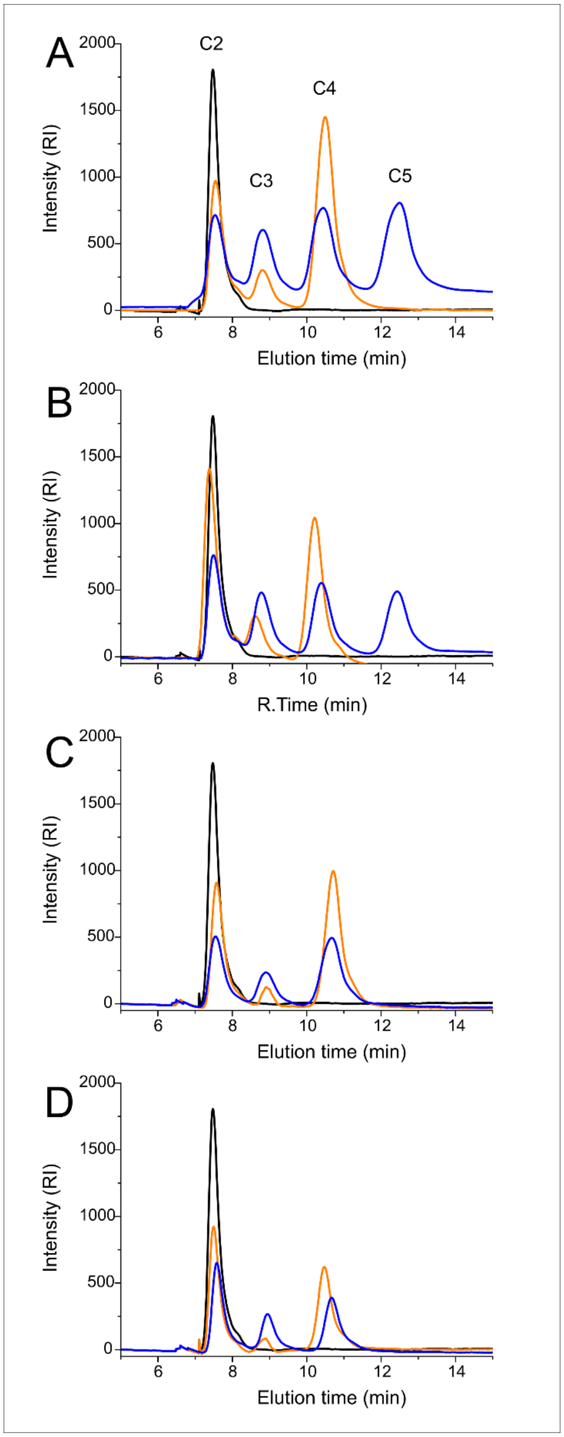

Supplementary Figure 3. Reaction time course analysis for cellodextrin synthesis by *Ral*CDP (blue), *Ral*ΔN63CDP (orange), and control (black): 4 h (A), 2 h (B), 1 h (C), and 30 min (D).
