# Supplementary material for: Insights to improve the activity of glycosyl phosphorylases from Ruminococcus albus 8 with cello-oligosaccharides
Source: Front Chem. 2023 Apr 7;11:1176537. doi: 10.3389/fchem.2023.1176537 (PMC10119399; doi:10.3389/fchem.2023.1176537)
Supplement: Supplementary file 4 [file Table2.DOCX]

Supplementary Material

Insights to improve the activity of glycosyl phosphorylases

from *Ruminococcus albus* 8 with cello oligosaccharides.

**Alem Storani ^1^, Sergio A. Guerrero ^1^, Alberto A Iglesias *^1^**

*** Correspondence:** Alberto A Iglesias: email: [iglesias@fbcb.unl.edu.ar](mailto:iglesias@fbcb.unl.edu.ar)

# Supplementary Figures and Tables

| **Enzyme** | **Microorganism** | **Substrate** | **Synthesis** | | | **Phosphorolysis** | | | **Author** |
| --- | --- | --- | --- | --- | --- | --- | --- | --- | --- |
|  |  |  | ***K*_M_**  **(mM)**  **(mg/ml)^a^** | ***k*_cat_**  **(s^-1^)** | ***k*_cat_/*K*_M_**  **(mM^-1^. s^-1^/**  **ml.mg^-1^.s^-1^)^a^** | ***K*_M_**  **(mM)**  **(mg/ml)^a^** | ***k*_cat_**  **(s^-1^)** | ***k*_cat_/*K*_M_**  **(mM^-1^. s^-1^/**  **ml.mg^-1^.s^-1^)^a^** |  |
| *Ral*CDP | *Ruminococcus albus* NE1 | **Glc-1P/Pi** | 166 | 11.8 | 0.07 | 181 | 39.6 | 0.2 | Sawano *et al.* 2013 |
|  |  | C2 | 13.2 | 47.1 | 3.6 | ND | ND | ND |  |
|  |  | C3 | 5 | 43.7 | 8.74 | 6.04 | 76.2 | 12.6 |  |
|  |  | C4 | 4 | 37.8 | 9.45 | 4.16 | 92.8 | 22.3 |  |
|  |  | C5 | 2.7 | 28.9 | 10.7 | 2.41 | 83.8 | 34.8 |  |
|  |  | C6 | 3.2 | 18.2 | 5.7 | 1.04 | 55.8 | 53.6 |  |
| *Ct*CDP | *Clostridium thermocellum* | C2 | 2.6 ± 0.2 | 17.0 ± 0.5 | 6.5 | - | - | - | O´neil *et al.* 2017 |
|  |  | C3 | 0.68 ± 0.08 | 9.5 ± 0.4 | 14 | - | - | - |  |
|  |  | C4 | 0.5 ± 0.1 | 5.0 ± 0.3 | 9.3 | - | - | - |  |
|  |  | C5 | 0.36 ± 0.08 | 4.3 ± 0.5 | 12 | - | - | - |  |
|  |  | C6 | 1.9 ± 0.8 | 7.6 ± 1.8 | 4 | - | - | - |  |
| *Ct*CDP | *Clostridium thermocellum* | C2 | 0.78 ± 0.02 | 2.67 ± 0.04 | 3.42 | ND | ND | ND | Ye *et al.* 2011 |
| *Ct*CDP-CBM3 |  |  | 0.59 ± 0.03 | 1.83 ± 0.08 | 3.1 | ND | ND | ND |  |
| *Ct*CDP-CBM4 |  |  | 0.53 ± 0.01 | 2.07 ± 0.08 | 3.9 | ND | ND | ND |  |
| *Ct*CDP-CBM6 |  |  | 0.76 ± 0.02 | 2.08 ± 0.05 | 2.73 | ND | ND | ND |  |
| *Ct*CDP-CBM9 |  |  | 1.08 ± 0.01 | 2.55 ± 0.02 | 2.36 | ND | ND | ND |  |
| CtCDP | *Clostridium thermocellum* | C6 | 1.29 ± 0.02 | 3.11 ± 0.05 | 2.41 | 1.82 ± 0.03 | 4.14 ± 0.10 | 2.27 |  |
| *Ct*CDP-CBM3 |  |  | 1.93 ± 0.04 | 1.08 ± 0.02 | 0.56 | 2.71 ± 0.02 | 2.70 ± 0.01 | 1 |  |
| *Ct*CDP-CBM4 |  |  | 0.29 ± 0.05 | 2.25 ± 0.05 | 7.76 | 1.56 ± 0.01 | 4.53 ± 0.03 | 2.9 |  |
| *Ct*CDP-CBM6 |  |  | 5.24 ± 0.05 | 2.16 ± 0.02 | 0.41 | 1.53 ± 0.01 | 1.37 ± 0.02 | 0.9 |  |
| *Ct*CDP-CBM9 |  |  | 1.46 ± 0.02 | 2.98 ± 0.04 | 2.04 | 0.62 ± 0.02 | 3.77 ± 0.06 | 6.08 |  |
| CDP/CBP | *Thermosipho africanus* strain TCF52B | **Glc-1P/Pi** | 1.57 | 7.61 | 4.85 | - | - | - | Wu *et al*. 2017 |
|  |  | Glc | 14.3 | 30.3 | 2.12 | - | - | - |  |
|  |  | C2 | 7.39 | 361 | 48.85 | 0.120 | 0.23 | 1.92 |  |
|  |  | C3 | 4.05 | 603 | 148.89 | 0.094 | 0.28 | 2.98 |  |
|  |  | C4 | 3.7 | 612 | 165.41 | 0.062 | 0.49 | 7.90 |  |
|  |  | C5 | 2.59 | 527 | 203.47 | 0.060 | 0.34 | 5.67 |  |
| *Ral*CDP | *R. albus* 8 | **Glc-1P/Pi** | 2.1 ± 0.1 | 310 ± 24 | 148 ± 18 | 0.5 ± 0.1 | 19 ± 1 | 39 ± 9 | This work |
|  |  | Glc | 0.8 ± 0.1 | 4.98 ± 0.08 | 6.2 ± 0.9 | - | - | - |  |
|  |  | C2 | 8.0 ± 1 | 310 ± 30 | 39 ± 8 | 4.3 ± 0.4 | 19.7 ± 0.4 | 4.6 ± 0.5 |  |
|  |  | C3 | 13 ± 3 | 720 ± 30 | 55 ± 15 | 1.9 ± 0.4 | 29.6 ± 0.5 | 16 ± 4 |  |
|  |  | C4 | 9 ± 3 | 1416 ± 38 | 157 ± 16 | 0.28 ± 0.08 | 28.2 ± 0.5 | 100 ± 3 |  |
|  |  | C5 | 1.5 ± 0.4 | 235 ± 9 | 156 ± 17 | 0.13 ± 0.03 | 20.4 ± 0.3 | 157 ± 4 |  |
|  |  | CMC | 6.5 ± 0.5 | 0.33 ± 0.03 | 0.05 ± 0.01 | 5.5 ± 0.4 | 0.024 ± 0.001 | (4.3±0.4)x10^-3^ |  |
|  |  | PASC | 8.4 ± 0.1 | 0.38 ± 0.03 | 0.05 ± 0.01 | 5.1 ± 0.8 | 0.028 ± 0.001 | (5 ± 1)x10^-3^ |  |
| *Ral*CDP-CBM37 | *R. albus* 8 | Glc | ND | ND | ND | - | - | - |  |
|  |  | C2 | 5.8 ± 0.8 | 149 ± 9 | 26 ± 5 | 11 ± 2 | 1.2 ± 0.1 | 0.11 ± 0.03 |  |
|  |  | C3 | 11 ± 2 | 757 ± 15 | 70 ± 10 | 2.2 ± 0.3 | 30.2 ± 0.4 | 14 ± 2 |  |
|  |  | C4 | 8 ± 1 | 1474 ± 28 | 180 ± 20 | 0.24 ± 0.06 | 27.5  ± 0.4 | 120 ± 20 |  |
|  |  | C5 | 1.3 ± 0.3 | 218 ± 6 | 170 ± 20 | 0.14 ± 0.03 | 16.4 ± 0.2 | 120 ± 20 |  |
|  |  | CMC | 3.0 ± 0.5 | 0.27 ± 0.03 | 0.09 ± 0.02 | 2.8 ± 0.3 | 0.28 ± 0.04 | 0.10 ± 0.02 |  |
|  |  | PASC | 2.1 ± 0.4 | 0.26 ± 0.02 | 0.12 ± 0.04 | 3.6 ± 0.7 | 0.27 ± 0.03 | 0.08 ± 0.02 |  |
| *Ral*∆N63CDP | *R. albus* 8 | **Glc-1P/Pi** | 0.32 ± 0..08 | 42 ± 5 | 131 ± 18 | 0.02 ± 0.01 | 16 ± 5 | (12 ± 4)x10^-4^ |  |
|  |  | Glc | 11 ± 2 | 0.47 ± 0.4 | 0.04 ± 0.01 | - | - | - |  |
|  |  | C2 | 22 ± 2 | 42 ± 5 | 1.91 ± 0.04 | 20 ± 3 | 0.02 ± 0.01 | (10± 4)x10^-4^ |  |
|  |  | C3 | 7 ± 2 | 0.02 ± 0.01 | (3 ± 1) x10^-3^ | ND | ND | ND |  |
|  |  | C4 | ND | ND | ND | ND | ND | ND |  |
|  |  | C5 | ND | ND | ND | ND | ND | ND |  |
|  |  | CMC | ND | ND | ND | ND | ND | ND |  |
|  |  | PASC | ND | ND | ND | ND | ND | ND |  |
| *Ral*CBP | *R. albus* 8 | **Glc-1P/Pi** | 0.58 ± 0.02 | 284 ± 1 | 489 ± 18 | 2.0 ± 0.2 | 178 ± 18 | 89 ± 9 |  |
|  |  | Glc | 2 ± 0.2 | 175 ± 2 | 88 ± 9 | - | - | - |  |
|  |  | C2 | 8 ± 2 | 0.41 ± 0.01 | 0.05 ± 0.01 | 2.4 ± 0.4 | 152 ± 4 | 63 ± 9 |  |
|  |  | C3 | 9.4 ± 0.6 | 0.14 ± 0.01 | 0.015 ± 0.002 | 5.2 ± 0.9 | 0.62 ± 0.01 | 0.12 ± 0.02 |  |
|  |  | C4 | ND | ND | ND | ND | ND | ND |  |
|  |  | C5 | ND | ND | ND | ND | ND | ND |  |
|  |  | CMC | ND | ND | ND | ND | ND | ND |  |
|  |  | PASC | ND | ND | ND | ND | ND | ND |  |

**Supplementary Table 2.** Kinetic parameters of previously characterized CDPs. Data for the comparative table was obtained from Ubiparip *et al*. 2021, Wu *et al*. 2017 and Ye *et al.* 2011. ^a^ units for substrates CMC and PASC; *ND: Not detected; -: Not measured
