# Supplementary material for: Insights to improve the activity of glycosyl phosphorylases from Ruminococcus albus 8 with cello-oligosaccharides
Source: Front Chem. 2023 Apr 7;11:1176537. doi: 10.3389/fchem.2023.1176537 (PMC10119399; doi:10.3389/fchem.2023.1176537)
Supplement: Supplementary file 5 [file DataSheet1.PDF]

Supplementary Material

Insights to improve the activity of glycosyl phosphorylases from *Ruminococcus albus* 8 with cello oligosaccharides.

Alem Storani [1], Sergio A. Guerrero [1], Alberto A Iglesias *[1]

*** Correspondence:** Alberto A Iglesias: email: iglesias@fbcb.unl.edu.ar

Supplementary Figures and Tables

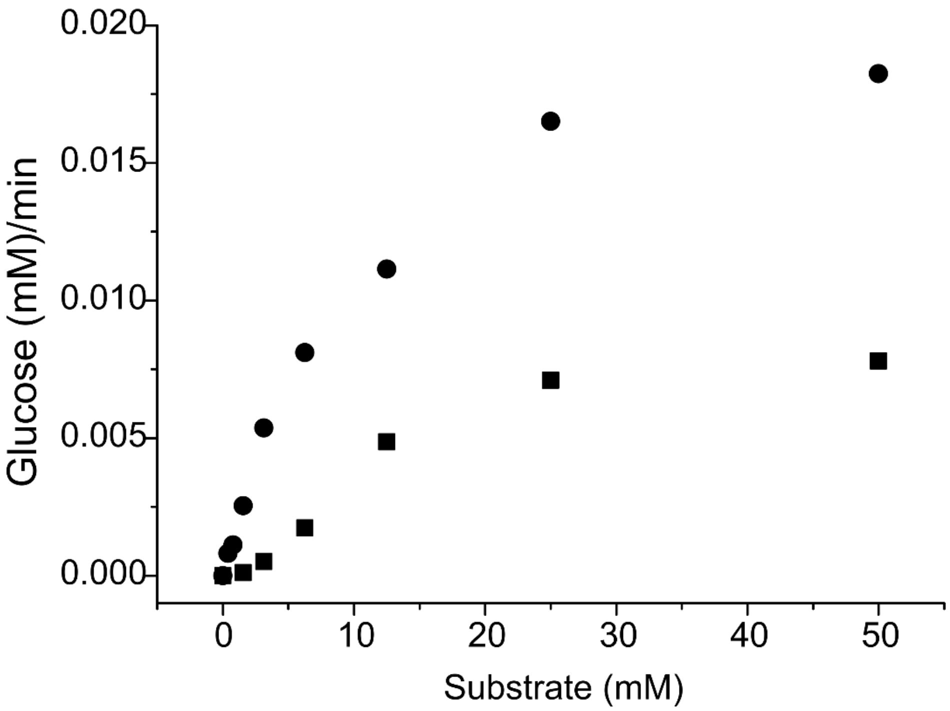

Supplementary Figure 1. Determination of kinetic constant of *Ral*CDP for phosphate and arsenate. The double rate of glucose appearance from arsenolysis vs. phosphorolysis correspond to the instability of glucose-1-arsenate and its instant conversion to glucose. *Ral*CDP affinity for each substrate was compared by determining K_M for phosphate (squares) and arsenate (circles) (K_M= 10.5± 0.1 mM and 11 ± 2 mM, respectively).
