# Supplementary material for: Insights to improve the activity of glycosyl phosphorylases from Ruminococcus albus 8 with cello-oligosaccharides
Source: Front Chem. 2023 Apr 7;11:1176537. doi: 10.3389/fchem.2023.1176537 (PMC10119399; doi:10.3389/fchem.2023.1176537)
Supplement: Supplementary file 6 [file Table3.DOCX]

Supplementary Material

Insights to improve the activity of glycosyl phosphorylases

from *Ruminococcus albus* 8 with cello oligosaccharides.

**Alem Storani ^1^, Sergio A. Guerrero ^1^, Alberto A Iglesias *^1^**

*** Correspondence:** Alberto A Iglesias: email: [iglesias@fbcb.unl.edu.ar](mailto:iglesias@fbcb.unl.edu.ar)

# Supplementary Figures and Tables

| **Enzyme** | **Substrate** | **Synthesis**  **(U/μmol)** | **Phosphorolysis**  **(U/μmol)** | **Microorganism** | **Author** |
| --- | --- | --- | --- | --- | --- |
| *Ct*CDP | RAC  (DP 14) | 5 | 14 | *Clostridium thermocellum* | Ye *et al.* 2011 |
| *Ct*CDP-CBM3 |  | 2 | 8 |  |  |
| *Ct*CDP-CBM4 |  | 5.5 | 17 |  |  |
| *Ct*CDP-CBM6 |  | 1 | 10 |  |  |
| *Ct*CDP-CBM9 |  | **18.9** | **5.9** |  |  |
| *Ct*CDP | RAC  (DP 164) | 2.5 | 0.6 |  |  |
| *Ct*CDP-CBM3 |  | 1.2 | 0.5 |  |  |
| *Ct*CDP-CBM4 |  | 2.5 | 0.8 |  |  |
| *Ct*CDP-CBM6 |  | 1 | 0.3 |  |  |
| *Ct*CDP-CBM9 |  | **4.1** | **1.2** |  |  |
| *Ral*CDP | PASC  (DP 132) | 22.8 | 1.68 | *Ruminococcus albus* | This work |
| *Ral*CDP-CBM37 |  | 15.6 | 16.2 |  |  |

**Supplementary Table 3.** Activity of CDP-CBM fusion proteins with long-chain cellulosic polysaccharides. PASC (Phosphoric Acid Swollen Cellulose) was prepared following the same steps as mentioned by Ye *et al.* 2011 for RAC DP 164 (Regenerated Amorphous Cellulose).
