# Supplementary material for: Insights to improve the activity of glycosyl phosphorylases from Ruminococcus albus 8 with cello-oligosaccharides
Source: Front Chem. 2023 Apr 7;11:1176537. doi: 10.3389/fchem.2023.1176537 (PMC10119399; doi:10.3389/fchem.2023.1176537)
Supplement: Supplementary file 7 [file Table4.DOCX]

Supplementary Material

Insights to improve the activity of glycosyl phosphorylases

from *Ruminococcus albus* 8 with cello oligosaccharides.

**Alem Storani ^1^, Sergio A. Guerrero ^1^, Alberto A Iglesias *^1^**

*** Correspondence:** Alberto A Iglesias: email: [iglesias@fbcb.unl.edu.ar](mailto:iglesias@fbcb.unl.edu.ar)

# Supplementary Figures and Tables

| **Enzyme** | **Substrate** | **A_max_**  **(μmol/g)** | ***K*_d_**  **(L/mol)** | **Microorganism** | **Author** |
| --- | --- | --- | --- | --- | --- |
| *Ct*CDP | RAC  (DP 14) | 0.44 ± 0.04 | 3.83 ± 0.35 | *Clostridium thermocellum* | Ye *et al.* 2011 |
| *Ct*CDP-CBM3 |  | 0.56 ± 0.03 | 1.51 ± 0.08 |  |  |
| *Ct*CDP-CBM4 |  | 0.32 ± 0.02 | 2.90 ± 0.18 |  |  |
| *Ct*CDP-CBM6 |  | 0.82 ± 0.06 | 1.03 ± 0.07 |  |  |
| *Ct*CDP-CBM9 |  | 2.55 ± 0.10 | 0.71 ± 0.03 |  |  |
| *Ct*CDP | RAC  (DP 164) | 0.52 ± 0.07 | 3.63 ± 0.49 |  |  |
| *Ct*CDP-CBM3 |  | 0.88 ± 0.05 | 1.5 ± 0.09 |  |  |
| *Ct*CDP-CBM4 |  | 2.92 ± 016 | 0.95 ± 0.05 |  |  |
| *Ct*CDP-CBM6 |  | 1.27 ± 0.09 | 1.45 ± 0.1 |  |  |
| *Ct*CDP-CBM9 |  | 4.93 ± 0.14 | 1.28 ± 0.04 |  |  |
| *Ral*CDP | PASC  (DP 132) | 0.50 ± 0.06 | 3.4 ± 0.4 | *Ruminococcus albus* 8 | This work |
| *Ral*CDP-CBM37 |  | 2.36 ± 0.05 | 1.73 ± 0.06 |  |  |

**Supplementary Table 4.** Langmuir isotherm parameters for the adsorption of CDP-CBM fusion proteins to acid treated amorphous cellulose.
